# Supplementary material for: Aboveground and belowground sizes are aligned in the unified spectrum of plant form and function
Source: Nat Commun. 2024 Oct 24;15:9199. doi: 10.1038/s41467-024-53180-x (PMC11502772; doi:10.1038/s41467-024-53180-x)
Supplement: Supplementary file 1 — Supplementary Information [file 41467_2024_53180_MOESM1_ESM.pdf]

Aboveground and belowground sizes are aligned in the unified spectrum of plant form and function

Eleonora Beccari[1*] & Carlos P. Carmona[1]

[1]Department of Botany, Institute of Ecology and Earth Sciences, University of Tartu, J. Liivi 2, 50409 Tartu, Estonia.

*Author for correspondence: Eleonora Beccari (eleonora.beccari@ut.ee; +39 3467923808)

Supplementary Figures and Tables

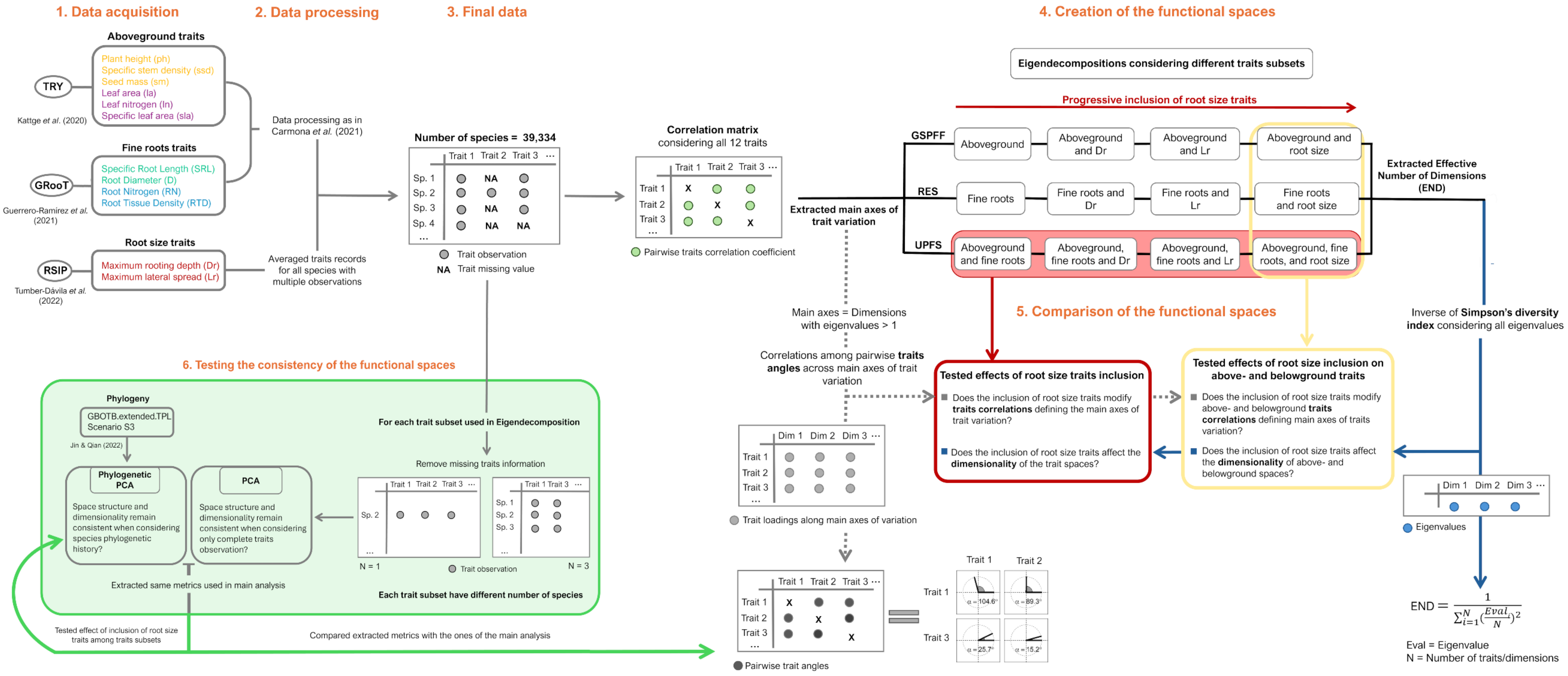

Supplementary Fig. 1 Methodological framework used in the analyses. Functional trait data were downloaded from three databases and aggregated into a single matrix of 39,334 species and twelve traits. For pairwise traits we extracted the correlation coefficients to build a trait-trait correlation matrix considering all twelve traits. We used the correlation matrix to perform a total of twelve eigendecompositions considering different traits subsets. For each eigendecomposition, we considered only the correlation coefficients of the considered traits. For each eigendecomposition, we extracted the main axes of trait variation that we used to calculate angles between pairs of traits in the considered dimensions. Furthermore, for each eigendecomposition, we extracted the

effective number of dimensions (END) by computing the inverse of Simpson's diversity index of dimensions' eigenvalues. To test the effect of the inclusion of root size traits among and within UPFS, GSPFF, and RES traits subsets we compared trait angles and END across eigendecompositions. Finally, we tested if results were consistent considering complete traits observations and after accounting for species non-independence. For each trait subset in the eigendecomposition we considered only species with complete observations (resulting in twelve trait matrices with different number of species depending on the subset of traits considered; Supplementary Table 1). For each trait subset we separately performed a Principal component analysis (PCA) and a phylogenetically informed principal component analysis from which we extracted trait angles and END. Then we compared these metrics within traits subsets progressively including root size traits and with the eigendecomposition outputs.

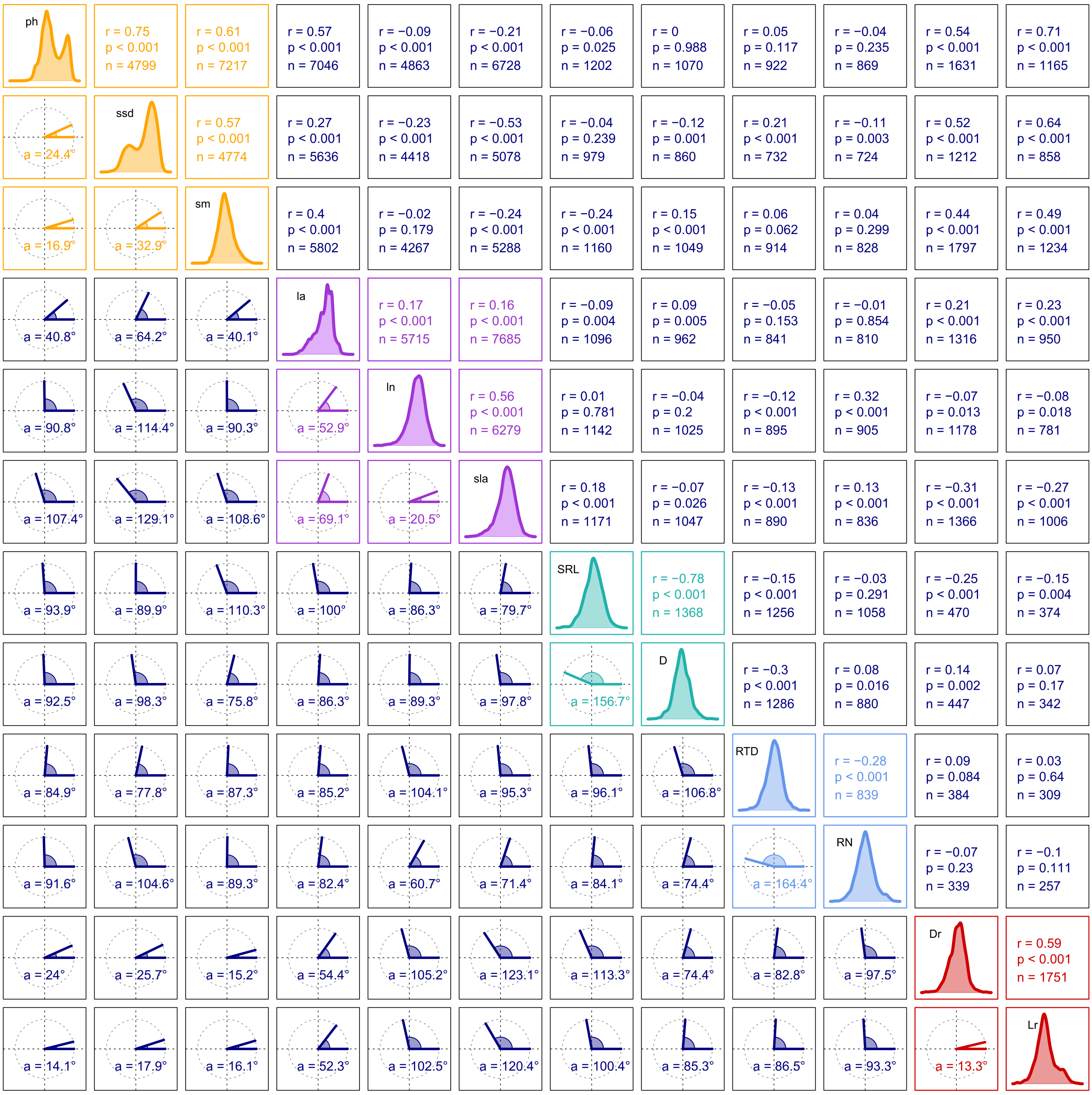

Supplementary Fig. 2 Traits distributions, correlations, and angles among traits in the eigenspace considering UPFS and root traits. Upper triangle shows the Pearson's correlation coefficient among pairs of traits (r), its two-sided p-value (p), and the number of species with observations for both traits. Diagonal shows the probabilistic distribution for each trait. Lower triangle shows the correlation among traits in the main eigenspace measured as angles between traits (a, angle in degrees among traits). *ph*, plant hight; *ssd*, specific steam density; *sm*, seed mass; *la*, leaf area; *ln*, leaf nitrogen; *sla*, specific leaf area; *SRL*, specific root length; *D*, root diameter; *RTD*, root tissue density; *RN*, root nitrogen; *Dr*, maximum rooting depth; *Lr*, maximum lateral root spread. Panels with the same colours shows the set of traits defining the four main axes of the UPFS and root size traits: *orange*, aboveground size (*i.e.*, ph, ssd, sm); *purple*, leaf economics spectrum (*i.e.*, la, ln, sla); *light green*, roots' collaboration gradient (*i.e.*, SRL, D); *light blue*, roots' economics spectrum (*i.e.*, RTD, RN); *red*, root size (*i.e.*, Dr, Lr).

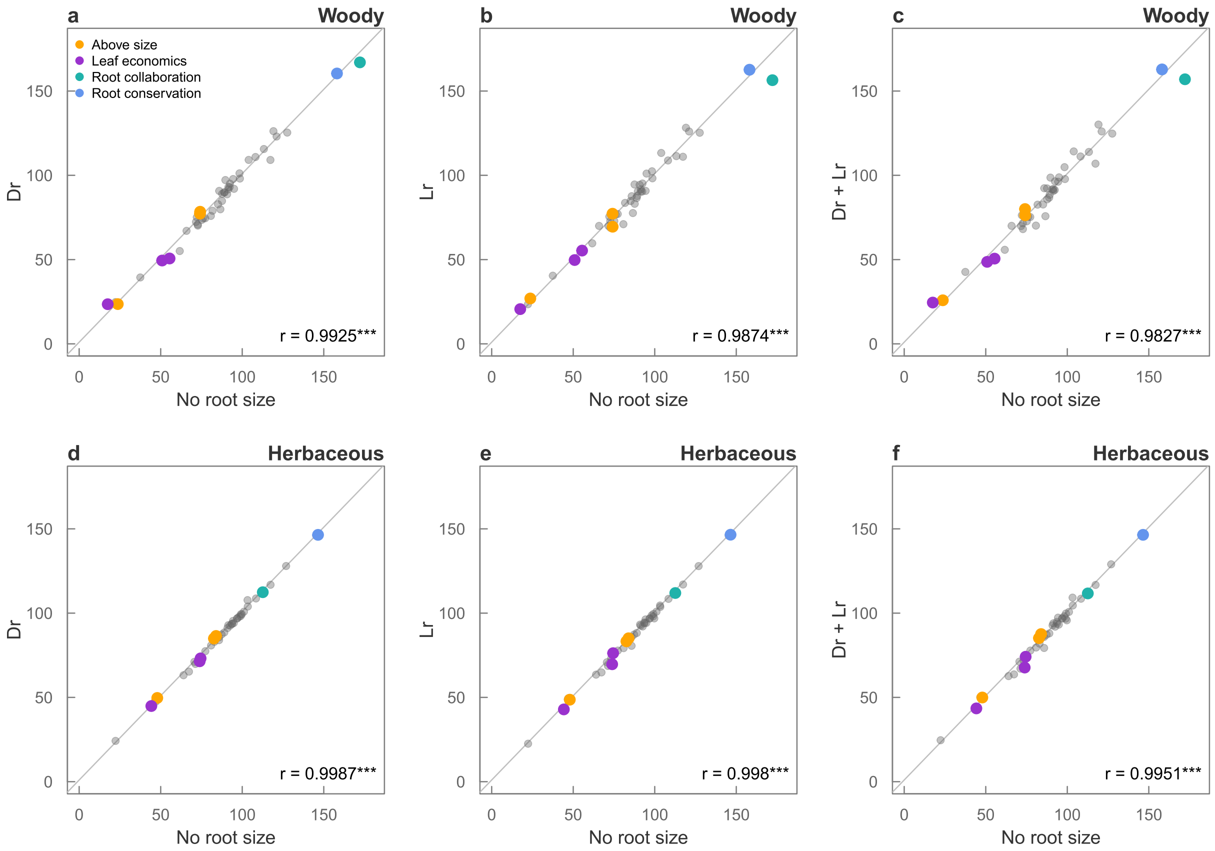

Supplementary Fig. 3 Correlations between angles in the UPFS within Woody and Herbaceous trait subsets progressively including root size traits. *No root size*, trait subsets excluding root size traits; *Dr*, traits subsets considering only rooting depth; *Lr*, traits subsets considering only lateral root spread; *Dr + Lr*, traits subsets considering both root size traits. Each dot represents an angle among pairwise variables. Coloured dots are the angles among highly correlated variable defining the main trade-offs present in across the UPFS, GSPFF, and RES: aboveground size traits (orange); leaf economics spectrum (purple); collaboration gradient (light green); and root conservation spectrum (light blue). Correlation coefficients (r) are in the lower part of each panel, together with their two-sided significance (*** p-value < 0.001). Exact p-value for each panel: **a** = $6.41e^{-41}$; **b** = $4.21e^{-36}$; **c** = $4.11e^{-33}$; **d** = $2.77e^{-57}$; **e** = $3.69e^{-53}$; **f** = $9.27e^{-45}$.

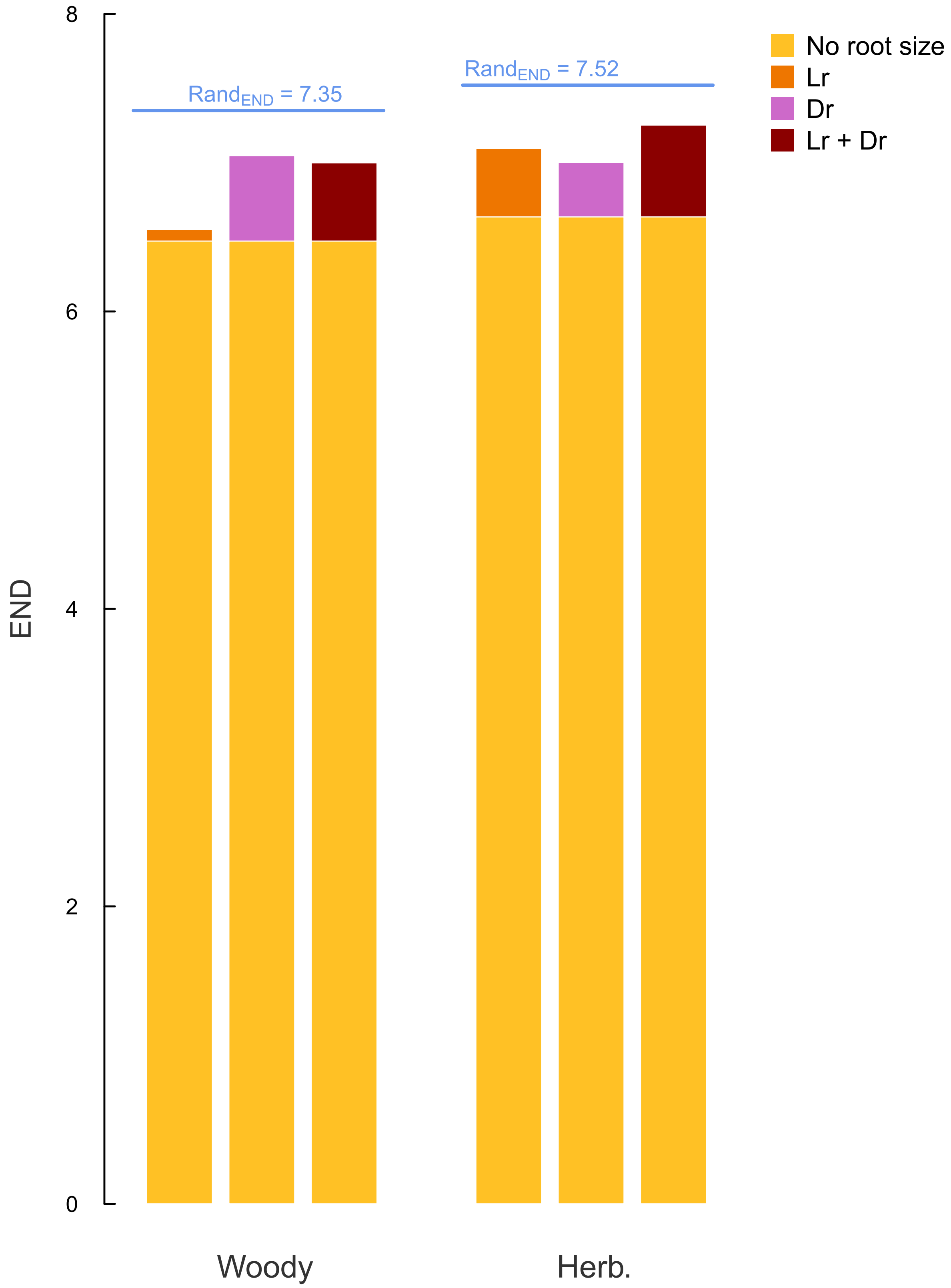

Supplementary Fig. 4 Effective number of dimensions (END) in the UPFS for Woody and Herbaceous (*Herb.*) species subsets considering different levels of root size inclusion. *No root size*, spaces excluding root size traits (yellow); *Lr*, spaces considering only lateral root spread (orange); *Dr*, spaces considering only rooting depth (pink); *Lr + Dr*, spaces considering both root size traits (dark red). Blue lines represent the END of dimension expected if we add a random uncorrelated trait to woody and herbaceous set of traits (*RandEND*).

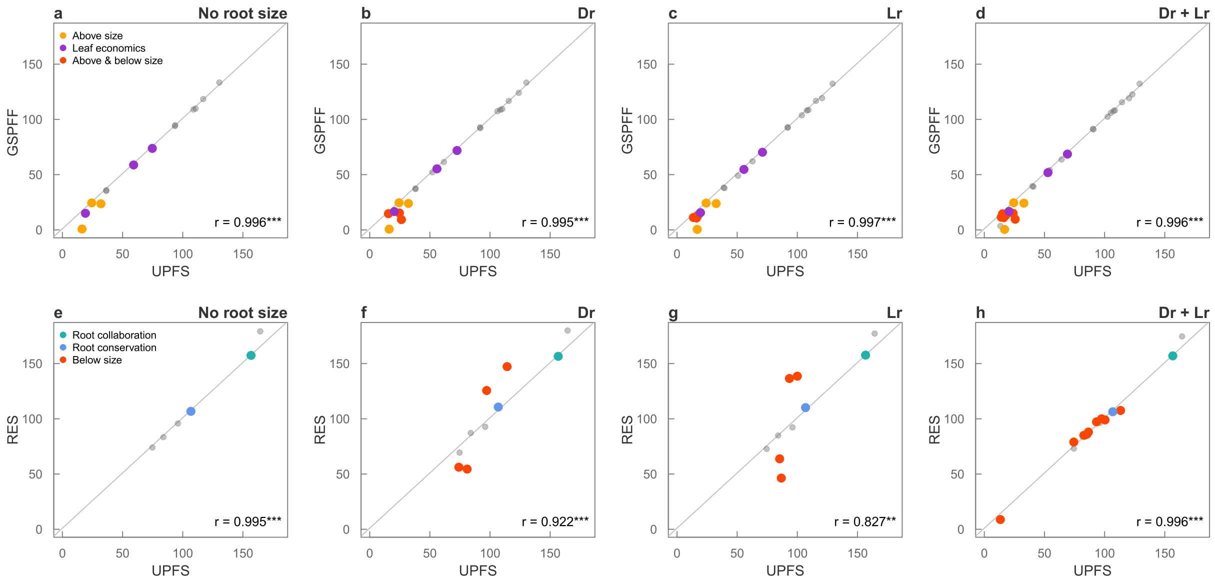

Supplementary Fig. 5 Correlations between angles across UPFS and both GSPFF, and RES traits subsets. *No root size*, traits subsets excluding root size traits; *Dr*, traits subsets considering only rooting depth; *Lr*, traits subsets considering only lateral root spread; *Dr + Lr*, traits subsets considering both lateral root spread and rooting depth. Coloured dots are the angles among highly correlated variable defining the main trade-offs present across the reduced spaces: (orange) angles among aboveground size traits (i.e., seed mass, specific stem density, and, plant hight); (purple) angles among traits defining the 'leaf economics spectrum' (i.e., leaf nitrogen, specific lean area, and leaf area); (light blue) angle between traits related to roots' 'conservation gradient' (i.e., root tissue density and root nitrogen); (light green) angle between traits defining the 'collaboration gradient' (i.e., specific root length and root diameter). In GSPFF reduced spaces, *Above & below size* label shows the angles among above- and belowground sizes (red). In RES reduced spaces *Below size* label shows the angles among belowground size and other root traits (red). Correlation coefficients (r) are in the lower part of each panel, together with their two-sided significance (*** p-value < 0.001; ** p-value < 0.01). Exact p-value for each panel: **a** = 2.41e^{-15}; **b** = 1.86e^{-20}; **c** = 1.08e^{-22}; **d** = 7.04e^{-29}; **e** = 3.94e^{-05}; **f** = 0.0001, **g** = 0.0031, **h** = 6.59e^{-15}.

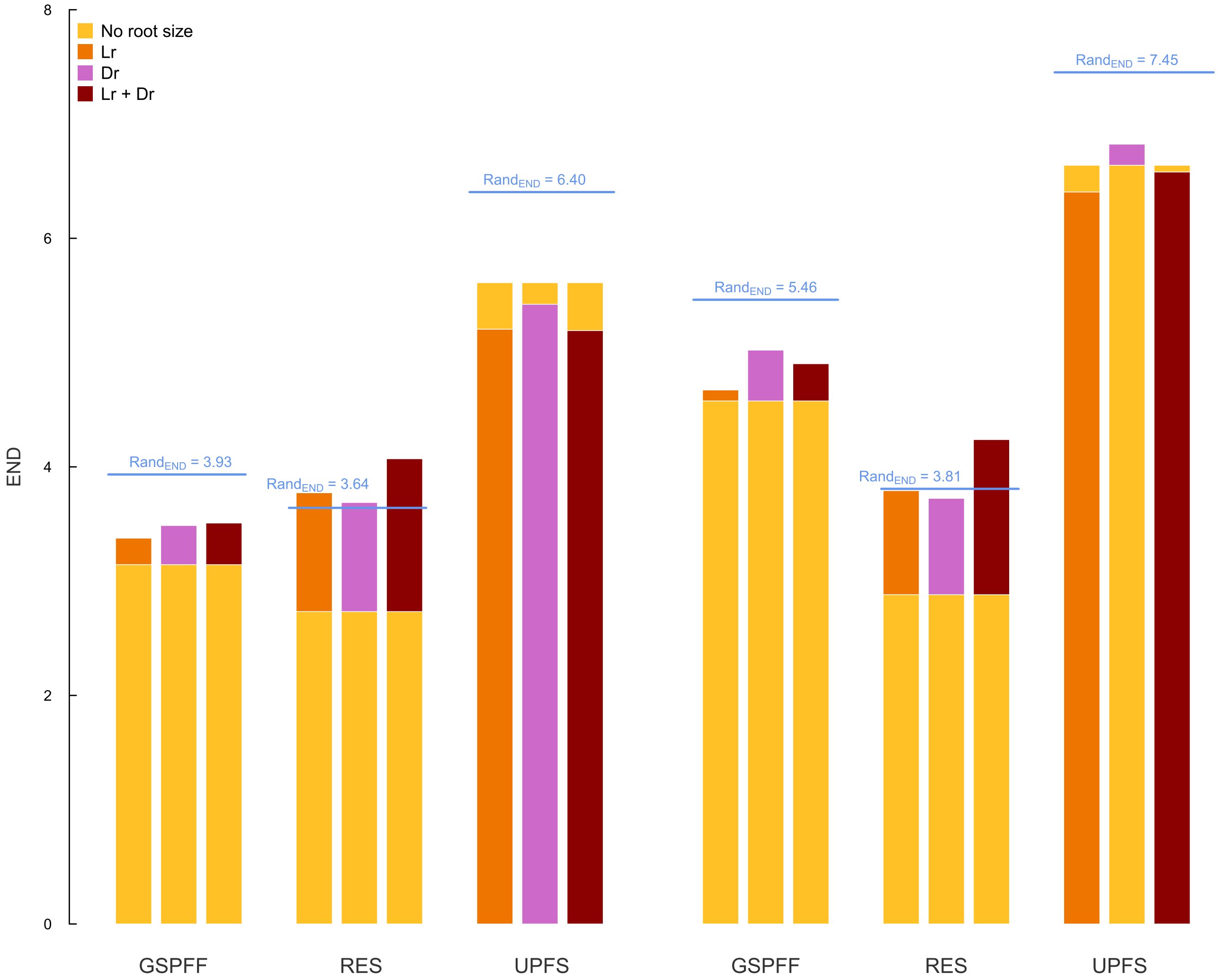

Supplementary Fig. 6 Effective number of dimensions (END) across GSPFF, RES, and UPFS traits subsets considering different levels of root size inclusion. The first three sets of histograms relate to PCA spaces considering only complete traits observations for all species; the second three sets of histograms relate to phylogenetically informed PCA built considering complete traits observations. *No root size*, spaces excluding root size traits (yellow); *Lr*, spaces considering only lateral root spread (orange); *Dr*, spaces considering only rooting depth (pink); *Lr + Dr*, spaces considering both root size traits (dark red). Blue lines represent the END of dimension expected if we add a random uncorrelated trait to the GSPFF, RES, and UPFS set of traits ($Rand_{END}$).

Supplementary Table 1 Summary statistics for each trait and trait subsets considered in the dataset. **a.** Summary statistics for each trait in the dataset. For each trait is shown the acronym used in the text (*Acronym*), its measurement unit (*Units*), both its mean and standard deviation (*Std. Dev.*), the number of species for which the trait has observations (*N. species*), and the relative coverage of the trait compared to the total number of species in the dataset (*Sp. Coverage*). Additionally, we show the number of families with information for each trait (*N. Families*) and their relative coverage (*Fam. Coverage*) relative to the total number of families in the dataset. We also report the number of Woody (*N. Woody*) and Herbaceous (*N. Herb*) for which the trait has observation together with the proportion of woody (*Prop. Woody*) and herbaceous (*Prop. Herb*) measurement relative to the number of species for which the trait had observations. In bold are showed the total number of species, the total number of families, the total number of woody and herbaceous species present in the dataset. It is important to note that woodiness information was available for 32,060 species. **b.** Summary statistics for species with complete trait information across the different trait subsets considered in the study. For each traits subset is shown the number of species with complete information for each trait subset (*N. species*), the number of families with complete information for each trait subset (*N. Families*), and the proportion of woody (*Prop. Woody*) and herbaceous (*Prop. Herb*) species relative to the number of species with complete information for the considered trait subset.

a.

Trait	Acronym	Units	Mean	Std. Dev.	N. Species	Sp. Coverage (%)	N. Families	Fam. Coverage (%)	N. Woody	N. Herb	Prop. Woody (%)	Prop. Herb (%)
Plant height	ph	m	5.75	9.52	13438	34.16	304	75.62	5842	5337	43.47	39.72
Specific stem density	ssd	g/m^3	0.49	0.23	11148	28.34	289	71.89	7757	2666	69.58	23.91
Seed mass	sm	mg	276.47	4594.96	23003	58.48	340	84.58	9440	9769	41.04	42.47
Leaf area	la	mm^2	5831.40	15079.64	12766	32.46	319	79.35	7755	3723	60.75	29.16
Leaf nitrogen concentration	ln	mg/g	21.08	8.63	9095	23.12	282	70.15	5719	2464	62.88	27.09
Specific leaf area	sla	mm^2/mg	17.40	10.93	9601	24.41	284	70.65	5004	3644	52.12	37.95
Specific root length	SRL	m/g	87.56	111.53	1697	4.31	154	38.31	777	752	45.79	44.31
Root diameter	D	mm	0.42	0.28	1550	3.94	147	36.57	712	695	45.94	44.84
Root tissue density	RTD	g/cm^3	0.30	0.18	1361	3.46	142	35.32	657	570	48.27	41.88
Root nitrogen concentration	RN	mg/g	14.20	7.49	1230	3.13	137	34.08	634	469	51.54	38.13
Maximum rooting depth	Dr	m	1.54	2.81	2598	6.60	184	45.77	923	1264	35.53	48.65
Maximum lateral root spread	Lr	m	1.56	3.57	1757	4.47	135	33.58	443	1017	25.21	57.88
				**Total**	**39334**		**402**		**19459**	**12601**	**No woodyness infos for 7274 species**	

b.

Traits subsets	N. Species	N. Families	Prop. Woody (%)	Prop. Herb (%)
UPFS + root size	134	31	27.61	70.90
UPFS + Dr	158	39	35.44	63.29
UPFS + Lr	135	32	28.15	70.37
UPFS	264	73	51.14	46.59
GSPFF+ root size	494	78	23.89	74.90
GSPFF + Dr	654	99	36.09	62.69
GSPFF + Lr	495	78	24.04	74.75
GSPFF	1734	158	49.08	48.96
RES + root size	151	32	29.14	69.54
RES + Dr	192	42	58.33	40.63
RES + Lr	152	33	29.61	69.08
RES	735	117	61.50	27.07

Supplementary Table 2 Eigendecompositions of the UPFS traits. ***UPFS and Dr+Lr*** refers to eigendecomposition performed on UPFS traits along with both Lr and Dr; ***UPFS***, considers only UPFS traits; ***UPFS and Dr***, considers UPFS traits and maximum rooting depth; ***UPFS and Lr***, considers UPFS traits and maximum lateral root spread. *Cum. variance,* cumulative variance explained by each axis; *N. of dimensions*, number of relevant dimensions retained; *Angles*, angles across pairs of traits considering all relevant dimensions; *END*, effective number of dimensions.

UPFS and Dr+Lr												
	PC1	*PC2*	*PC3*	*PC4*	*PC5*	*PC6*	*PC7*	*PC8*	*PC9*	*PC10*	*PC11*	*PC12*
Eigenvalues	3.86	1.94	1.77	1.11	0.90	0.73	0.46	0.43	0.38	0.22	0.12	0.09
Cum. variance	32.14	48.33	63.05	72.31	79.77	85.83	89.69	93.25	96.42	98.23	99.27	100.00
Loadings	*PC1*	*PC2*	*PC3*	*PC4*	*PC5*	*PC6*	*PC7*	*PC8*	*PC9*	*PC10*	*PC11*	*PC12*
la	-0.44	-0.37	0.44	0.27	0.41	-0.35	0.28	-0.05	0.11	0.15	-0.08	-0.01
ln	0.24	-0.54	0.53	0.22	-0.33	0.22	-0.16	-0.08	0.36	0.01	0.05	-0.03
ph	-0.86	-0.05	0.32	-0.02	0.12	-0.05	0.06	0.19	-0.04	-0.16	0.25	0.00
sla	0.50	-0.41	0.51	0.33	0.12	0.19	-0.03	0.11	-0.33	-0.17	-0.09	0.04
ssd	-0.86	0.26	0.11	-0.11	-0.11	-0.10	-0.06	0.08	0.20	-0.26	-0.19	0.01
sm	-0.75	-0.20	0.11	0.02	-0.07	-0.26	-0.43	-0.28	-0.22	0.06	0.00	0.00
SRL	0.29	0.54	0.66	-0.33	0.15	0.05	-0.01	-0.09	-0.05	0.00	-0.01	-0.19
D	-0.15	-0.70	-0.64	0.00	0.18	0.03	-0.03	0.06	0.00	-0.09	-0.02	-0.19
RTD	-0.16	0.46	-0.08	0.71	-0.43	-0.16	0.09	0.09	-0.09	0.02	0.01	-0.10
RN	0.14	-0.48	0.20	-0.49	-0.55	-0.30	0.20	0.12	-0.13	0.03	-0.02	-0.01
Dr	-0.73	-0.07	-0.04	-0.01	-0.17	0.42	0.33	-0.37	-0.11	-0.03	-0.01	0.00
Lr	-0.80	-0.02	0.11	-0.10	-0.03	0.38	-0.11	0.34	-0.06	0.25	-0.06	-0.02
Angles	***la***	***ln***	***ph***	***sla***	***ssd***	***sm***	***SRL***	***D***	***RTD***	***N***	***Dr***	***Lr***
la	0.00	52.90	40.78	69.12	64.19	40.05	99.95	86.32	85.19	82.43	54.36	52.25
ln	52.90	0.00	90.76	20.54	114.42	90.26	86.30	89.34	104.06	60.71	105.17	102.50
ph	40.78	90.76	0.00	107.44	24.45	16.85	93.94	92.45	84.90	91.57	23.98	14.08
sla	69.12	20.54	107.44	0.00	129.09	108.56	79.67	97.75	95.30	71.39	123.10	120.38
ssd	64.19	114.42	24.45	129.09	0.00	32.90	89.88	98.25	77.77	104.62	25.67	17.86
sm	40.05	90.26	16.85	108.56	32.90	0.00	110.30	75.85	87.29	89.29	15.19	16.13
SRL	99.95	86.30	93.94	79.67	89.88	110.30	0.00	156.67	96.10	84.15	113.26	100.38
D	86.32	89.34	92.45	97.75	98.25	75.85	156.67	0.00	106.77	74.43	74.40	85.28
RTD	85.19	104.06	84.90	95.30	77.77	87.29	96.10	106.77	0.00	164.38	82.77	86.54
RN	82.43	60.71	91.57	71.39	104.62	89.29	84.15	74.43	164.38	0.00	97.49	93.30
Dr	54.36	105.17	23.98	123.10	25.67	15.19	113.26	74.40	82.77	97.49	0.00	13.35
Lr	52.25	102.50	14.08	120.38	17.86	16.13	100.38	85.28	86.54	93.30	13.35	0.00
N. of dimensions	4											
END	5.77											

UPFS										
	PC1	*PC2*	*PC3*	*PC4*	*PC5*	*PC6*	*PC7*	*PC8*	*PC9*	*PC10*
Eigenvalues	2.88	1.94	1.75	1.10	0.88	0.49	0.40	0.33	0.13	0.09
Cum. variance	28.78	48.13	65.67	76.72	85.56	90.46	94.51	97.77	99.12	100.00
Loadings	*PC1*	*PC2*	*PC3*	*PC4*	*PC5*	*PC6*	*PC7*	*PC8*	*PC9*	*PC10*
la	-0.48	-0.40	0.47	-0.22	0.35	0.37	0.05	0.27	-0.05	0.00
ln	0.32	-0.52	0.52	-0.23	-0.29	-0.34	0.28	0.15	0.04	-0.04
ph	-0.84	-0.10	0.35	0.05	0.09	0.04	0.12	-0.29	0.22	-0.02
sla	0.55	-0.38	0.49	-0.35	0.16	0.00	-0.19	-0.33	-0.11	0.04
ssd	-0.87	0.21	0.14	0.14	-0.14	-0.12	0.22	-0.12	-0.25	0.02
sm	-0.77	-0.25	0.15	0.02	-0.13	-0.28	-0.46	0.12	0.00	0.00
SRL	0.30	0.56	0.64	0.33	0.17	-0.03	-0.08	0.02	-0.02	-0.19
D	-0.14	-0.71	-0.63	-0.01	0.17	-0.03	0.03	-0.09	-0.05	-0.19
RTD	-0.21	0.44	-0.07	-0.71	-0.45	0.19	-0.06	-0.02	0.00	-0.10
RN	0.17	-0.47	0.20	0.50	-0.59	0.32	-0.07	-0.04	-0.02	-0.01
Angles	***la***	***ln***	***ph***	***sla***	***ssd***	***sm***	***SRL***	***D***	***RTD***	***N***
la	0.00	59.20	36.43	74.72	60.10	36.50	100.14	85.62	86.13	81.15
ln	59.20	0.00	93.50	19.14	117.01	93.69	85.76	90.01	103.48	62.24
ph	36.43	93.50	0.00	108.91	24.36	16.32	94.01	92.21	84.20	90.32
sla	74.72	19.14	108.91	0.00	130.46	110.72	79.82	97.82	95.10	73.00
ssd	60.10	117.01	24.36	130.46	0.00	32.02	89.94	97.92	77.27	103.05
sm	36.50	93.69	16.32	110.72	32.02	0.00	109.63	75.99	87.40	87.65
SRL	100.14	85.76	94.01	79.82	89.94	109.63	0.00	156.79	96.02	83.95
D	85.62	90.01	92.21	97.82	97.92	75.99	156.79	0.00	106.80	74.79
RTD	86.13	103.48	84.20	95.10	77.27	87.40	96.02	106.80	0.00	164.45
RN	81.15	62.24	90.32	73.00	103.05	87.65	83.95	74.79	164.45	0.00
N. of dimensions	4									
END	5.67									

UPFS and Dr											
	PC1	*PC2*	*PC3*	*PC4*	*PC5*	*PC6*	*PC7*	*PC8*	*PC9*	*PC10*	*PC11*
Eigenvalues	3.30	1.94	1.75	1.10	0.90	0.63	0.46	0.38	0.31	0.13	0.09
Cum. variance	29.97	47.62	63.57	73.61	81.76	87.51	91.70	95.17	98.00	99.20	100.00
Loadings	*PC1*	*PC2*	*PC3*	*PC4*	*PC5*	*PC6*	*PC7*	*PC8*	*PC9*	*PC10*	*PC11*
la	-0.46	0.37	0.48	-0.22	0.39	-0.26	0.27	-0.07	0.26	-0.06	0.00
ln	0.28	0.54	0.52	-0.23	-0.32	0.22	-0.19	-0.35	0.06	0.05	-0.04
ph	-0.84	0.06	0.36	0.04	0.11	-0.02	0.10	-0.02	-0.29	0.23	-0.01
sla	0.53	0.40	0.49	-0.35	0.13	0.10	0.00	0.28	-0.26	-0.11	0.04
ssd	-0.85	-0.25	0.15	0.14	-0.11	-0.03	-0.05	-0.23	-0.20	-0.24	0.02
sm	-0.76	0.21	0.16	0.01	-0.08	-0.16	-0.48	0.26	0.13	0.01	0.00
SRL	0.32	-0.55	0.64	0.33	0.15	0.08	-0.04	0.07	0.04	-0.02	-0.19
D	-0.17	0.71	-0.62	-0.01	0.18	0.00	-0.01	-0.02	-0.10	-0.05	-0.19
RTD	-0.19	-0.45	-0.07	-0.71	-0.43	-0.19	0.12	0.08	-0.01	0.01	-0.10
RN	0.15	0.48	0.20	0.50	-0.56	-0.26	0.23	0.12	-0.01	-0.02	-0.01
Dr	-0.71	0.08	-0.02	0.01	-0.16	0.60	0.20	0.18	0.14	-0.03	0.00
Angles	***la***	***ln***	***ph***	***sla***	***ssd***	***sm***	***SRL***	***D***	***RTD***	***N***	***Dr***
la	0.00	55.87	37.97	72.61	61.67	38.10	99.23	86.66	86.15	80.78	52.08
ln	55.87	0.00	91.92	20.46	115.57	91.74	86.56	89.25	103.52	62.13	106.21
ph	37.97	91.92	0.00	108.60	24.42	16.21	93.92	92.64	84.15	90.22	24.70
sla	72.61	20.46	108.60	0.00	130.23	110.16	79.97	97.52	95.10	73.08	123.90
ssd	61.67	115.57	24.42	130.23	0.00	32.19	89.82	98.38	77.24	102.93	26.32
sm	38.10	91.74	16.21	110.16	32.19	0.00	109.41	76.52	87.43	87.40	15.54
SRL	99.23	86.56	93.92	79.97	89.82	109.41	0.00	156.70	96.03	84.03	114.20
D	86.66	89.25	92.64	97.52	98.38	76.52	156.70	0.00	106.84	74.68	74.11
RTD	86.15	103.52	84.15	95.10	77.24	87.43	96.03	106.84	0.00	164.48	81.00
RN	80.78	62.13	90.22	73.08	102.93	87.40	84.03	74.68	164.48	0.00	97.22
Dr	52.08	106.21	24.70	123.90	26.32	15.54	114.20	74.11	81.00	97.22	0.00
N. of dimensions	4										
END	5.87										

UPFS and Lr											
	PC1	*PC2*	*PC3*	*PC4*	*PC5*	*PC6*	*PC7*	*PC8*	*PC9*	*PC10*	*PC11*
Eigenvalues	3.40	1.94	1.76	1.11	0.88	0.64	0.45	0.38	0.22	0.12	0.09
Cum. variance	30.93	48.54	64.58	74.68	82.72	88.53	92.60	96.08	98.07	99.21	100.00
Loadings	*PC1*	*PC2*	*PC3*	*PC4*	*PC5*	*PC6*	*PC7*	*PC8*	*PC9*	*PC10*	*PC11*
la	0.47	0.40	0.42	-0.26	0.34	0.43	-0.16	0.13	0.15	-0.08	0.01
ln	-0.27	0.54	0.53	-0.22	-0.29	-0.28	0.14	0.36	0.00	0.05	0.03
ph	0.88	0.09	0.31	0.02	0.09	-0.01	-0.17	-0.07	-0.16	0.25	0.00
sla	-0.50	0.40	0.51	-0.34	0.16	-0.15	-0.04	-0.34	-0.17	-0.09	-0.04
ssd	0.87	-0.23	0.10	0.11	-0.14	-0.02	-0.02	0.17	-0.27	-0.19	-0.01
sm	0.76	0.23	0.10	-0.02	-0.13	0.14	0.53	-0.20	0.06	0.00	0.00
SRL	-0.27	-0.54	0.67	0.33	0.17	0.02	0.08	-0.04	0.00	-0.01	0.19
D	0.13	0.69	-0.65	0.00	0.17	-0.05	-0.02	-0.01	-0.10	-0.02	0.19
RTD	0.17	-0.45	-0.08	-0.72	-0.45	0.09	-0.12	-0.10	0.02	0.01	0.10
RN	-0.15	0.48	0.20	0.49	-0.59	0.23	-0.21	-0.14	0.03	-0.02	0.01
Lr	0.78	0.04	0.10	0.10	0.01	-0.51	-0.16	-0.12	0.24	-0.07	0.02
Angles	***la***	***ln***	***ph***	***sla***	***ssd***	***sm***	***SRL***	***D***	***RTD***	***N***	***Lr***
la	0.00	55.62	39.51	71.03	62.75	38.72	100.82	85.31	85.44	82.63	50.69
ln	55.62	0.00	92.12	19.51	115.50	91.93	85.59	90.07	103.86	60.83	103.76
ph	39.51	92.12	0.00	107.89	24.24	16.82	94.21	91.87	85.13	91.67	13.60
sla	71.03	19.51	107.89	0.00	129.34	109.20	79.51	98.07	95.14	71.33	120.58
ssd	62.75	115.50	24.24	129.34	0.00	32.57	90.17	97.63	78.03	104.65	17.36
sm	38.72	91.93	16.82	109.20	32.57	0.00	110.55	75.29	87.53	89.47	16.25
SRL	100.82	85.59	94.21	79.51	90.17	110.55	0.00	156.74	95.99	84.05	100.02
D	85.31	90.07	91.87	98.07	97.63	75.29	156.74	0.00	106.85	74.55	85.34
RTD	85.44	103.86	85.13	95.14	78.03	87.53	95.99	106.85	0.00	164.30	86.68
RN	82.63	60.83	91.67	71.33	104.65	89.47	84.05	74.55	164.30	0.00	93.47
Lr	50.69	103.76	13.60	120.58	17.36	16.25	100.02	85.34	86.68	93.47	0.00
N. of dimensions	4										
END	5.68										

Supplementary Table 3 Eigendecompositions of the **Woody species** subset (N = 19,459) considering UPFS traits while progressively including root size traits. ***UPFS and Dr+Lr*** refers to eigendecomposition performed on UPFS traits along with both Lr and Dr; ***UPFS***, considers only UPFS traits; ***UPFS and Dr***, considers UPFS traits and maximum rooting depth; ***UPFS and Lr***, considers UPFS traits and maximum lateral root spread. *Cum. variance*, cumulative variance explained by each axis; *N. of dimensions*, number of relevant dimensions retained; *Angles*, angles across pairs of traits considering all relevant dimensions; *END*, effective number of dimensions.

Woody

UPFS and Dr+Lr												
	PC1	*PC2*	*PC3*	*PC4*	*PC5*	*PC6*	*PC7*	*PC8*	*PC9*	*PC10*	*PC11*	*PC12*
Eigenvalues	2.95	2.07	1.94	1.13	0.95	0.79	0.64	0.49	0.41	0.31	0.25	0.05
Cum. variance	24.60	41.85	58.03	67.46	75.40	82.02	87.36	91.44	94.89	97.51	99.59	100.00
Loadings	*PC1*	*PC2*	*PC3*	*PC4*	*PC5*	*PC6*	*PC7*	*PC8*	*PC9*	*PC10*	*PC11*	*PC12*
la	-0.75	-0.15	-0.15	0.09	0.11	-0.35	0.15	-0.30	0.26	0.25	-0.07	0.00
ln	-0.43	-0.04	-0.64	-0.16	-0.41	0.24	0.11	0.16	-0.18	0.28	0.10	-0.03
ph	-0.81	-0.23	0.21	0.04	0.14	-0.11	0.01	-0.17	-0.26	-0.16	0.30	-0.03
sla	-0.37	-0.17	-0.74	0.15	-0.21	0.11	0.17	0.00	0.22	-0.36	-0.03	0.01
ssd	0.00	-0.23	0.47	-0.64	-0.11	-0.01	0.54	0.02	0.00	-0.06	-0.06	0.01
sm	-0.63	0.09	0.31	-0.03	-0.23	-0.43	-0.17	0.47	0.11	-0.04	-0.01	0.00
SRL	0.15	-0.79	-0.32	-0.22	0.37	-0.08	-0.13	0.13	-0.02	-0.01	-0.11	-0.13
D	-0.21	0.91	0.08	0.17	0.06	0.04	0.21	-0.01	-0.04	-0.05	-0.10	-0.15
RTD	0.15	-0.46	0.40	0.24	-0.68	-0.04	-0.13	-0.22	-0.07	-0.03	-0.12	-0.06
RN	-0.03	0.44	-0.45	-0.57	-0.12	-0.31	-0.26	-0.21	-0.19	-0.09	-0.12	0.02
Dr	-0.53	0.11	0.32	-0.42	-0.02	0.47	-0.34	-0.09	0.28	0.00	0.04	-0.03
Lr	-0.81	-0.16	0.17	0.17	0.18	0.27	-0.02	0.05	-0.25	-0.01	-0.30	0.06
Angles	***la***	***ln***	***ph***	***sla***	***ssd***	***sm***	***SRL***	***D***	***RTD***	***N***	***Dr***	***Lr***
la	0.00	48.65	25.77	50.57	98.63	42.67	86.73	88.41	98.80	92.34	60.20	22.80
ln	48.65	0.00	71.59	24.51	106.92	82.69	72.74	91.79	130.11	55.79	81.99	71.14
ph	25.77	71.59	0.00	75.37	79.98	25.86	90.77	91.31	82.59	104.86	45.72	10.56
sla	50.57	24.51	75.37	0.00	124.74	91.65	68.11	97.80	114.18	75.26	101.02	72.06
ssd	98.63	106.92	79.98	124.74	0.00	76.29	76.29	111.17	75.76	85.74	51.52	89.85
sm	42.67	82.69	25.86	91.65	76.29	0.00	113.88	69.88	92.23	96.21	31.59	26.66
SRL	86.73	72.74	90.77	68.11	76.29	113.88	0.00	162.83	70.16	96.39	105.23	96.37
D	88.41	91.79	91.31	97.80	111.17	69.88	162.83	0.00	126.05	70.02	76.66	85.21
RTD	98.80	130.11	82.59	114.18	75.76	92.23	70.16	126.05	0.00	156.97	101.77	84.31
RN	92.34	55.79	104.86	75.26	85.74	96.21	96.39	70.02	156.97	0.00	75.87	107.05
Dr	60.20	81.99	45.72	101.02	51.52	31.59	105.23	76.66	101.77	75.87	0.00	52.27
Lr	22.80	71.14	10.56	72.06	89.85	26.66	96.37	85.21	84.31	107.05	52.27	0.00
N. of dimensions	4											
END	7.00											

UPFS										
	PC1	*PC2*	*PC3*	*PC4*	*PC5*	*PC6*	*PC7*	*PC8*	*PC9*	*PC10*
Eigenvalues	2.32	2.03	1.76	1.03	0.92	0.67	0.49	0.39	0.31	0.08
Cum. variance	23.22	43.55	61.12	71.38	80.55	87.23	92.12	96.04	99.17	100.00
Loadings	*PC1*	*PC2*	*PC3*	*PC4*	*PC5*	*PC6*	*PC7*	*PC8*	*PC9*	*PC10*
la	-0.74	0.29	-0.27	0.01	-0.23	-0.02	0.30	0.29	0.26	0.01
ln	-0.62	0.22	0.41	-0.03	0.46	-0.18	-0.16	-0.23	0.27	-0.03
ph	-0.58	0.30	-0.55	0.06	-0.20	-0.03	0.16	-0.41	-0.19	0.00
sla	-0.61	0.34	0.47	-0.26	0.12	-0.20	-0.02	0.22	-0.35	0.02
ssd	0.23	0.18	-0.42	0.64	0.34	-0.43	0.02	0.12	-0.06	0.00
sm	-0.50	-0.03	-0.62	0.06	0.14	0.34	-0.45	0.14	-0.04	0.00
SRL	0.18	0.80	0.31	0.27	-0.30	0.14	-0.12	0.02	-0.01	-0.18
D	-0.33	-0.89	-0.09	-0.06	-0.06	-0.19	0.03	0.04	-0.05	-0.20
RTD	0.36	0.36	-0.39	-0.43	0.54	0.19	0.24	0.04	-0.02	-0.10
RN	-0.34	-0.31	0.42	0.52	0.27	0.45	0.26	-0.01	-0.09	0.01
Angles	***la***	***ln***	***ph***	***sla***	***ssd***	***sm***	***SRL***	***D***	***RTD***	***N***
la	0.00	50.87	22.22	55.43	89.67	37.49	88.90	89.21	94.97	85.88
ln	50.87	0.00	72.73	17.52	117.28	85.18	75.18	91.50	119.13	61.68
ph	22.22	72.73	0.00	76.09	74.10	23.68	91.50	92.31	81.80	98.32
sla	55.43	17.52	76.09	0.00	127.60	90.71	72.79	98.58	103.95	77.28
ssd	89.67	117.28	74.10	127.60	0.00	74.18	72.15	108.13	86.60	87.71
sm	37.49	85.18	23.68	90.71	74.18	0.00	113.27	71.64	87.59	94.40
SRL	88.90	75.18	91.50	72.79	72.15	113.27	0.00	158.11	80.77	92.47
D	89.21	91.50	92.31	98.58	108.13	71.64	158.11	0.00	121.07	65.84
RTD	94.97	119.13	81.80	103.95	86.60	87.59	80.77	121.07	0.00	172.17
RN	85.88	61.68	98.32	77.28	87.71	94.40	92.47	65.84	172.17	0.00
N. of dimensions	4									
END	6.47									

UPFS and Dr											
	PC1	*PC2*	*PC3*	*PC4*	*PC5*	*PC6*	*PC7*	*PC8*	*PC9*	*PC10*	*PC11*
Eigenvalues	2.45	2.04	1.89	1.11	0.93	0.71	0.64	0.49	0.36	0.31	0.08
Cum. variance	22.23	40.76	57.91	67.98	76.42	82.89	88.71	93.15	96.44	99.29	100.00
Loadings	*PC1*	*PC2*	*PC3*	*PC4*	*PC5*	*PC6*	*PC7*	*PC8*	*PC9*	*PC10*	*PC11*
la	-0.73	0.33	0.10	0.17	0.27	0.13	-0.15	-0.27	0.26	0.26	0.01
ln	-0.52	0.31	-0.47	-0.11	-0.47	-0.11	-0.11	0.15	-0.23	0.27	-0.03
ph	-0.66	0.28	0.44	0.09	0.22	-0.03	0.00	-0.18	-0.40	-0.18	0.00
sla	-0.45	0.46	-0.55	0.18	-0.19	-0.15	-0.16	0.03	0.21	-0.35	0.02
ssd	0.10	0.09	0.54	-0.59	-0.16	0.06	-0.55	0.01	0.06	-0.06	0.00
sm	-0.61	-0.05	0.46	0.10	-0.07	0.39	0.15	0.47	0.08	-0.04	0.00
SRL	0.28	0.81	-0.13	-0.27	0.34	0.06	0.13	0.12	0.02	-0.01	-0.18
D	-0.38	-0.85	-0.14	0.14	0.06	-0.05	-0.20	-0.01	0.00	-0.05	-0.19
RTD	0.31	0.29	0.47	0.35	-0.61	0.16	0.11	-0.24	0.04	-0.02	-0.09
RN	-0.29	-0.23	-0.47	-0.53	-0.13	0.47	0.23	-0.26	-0.01	-0.09	0.01
Dr	-0.47	-0.11	0.41	-0.40	-0.13	-0.50	0.37	-0.04	0.16	0.00	-0.02
Angles	***la***	***ln***	***ph***	***sla***	***ssd***	***sm***	***SRL***	***D***	***RTD***	***N***	***Dr***
la	0.00	49.44	24.77	50.66	97.18	39.40	89.91	89.81	91.98	90.74	63.00
ln	49.44	0.00	70.79	23.52	109.11	82.80	73.55	91.69	126.19	55.07	83.21
ph	24.77	70.79	0.00	74.42	77.22	23.60	92.96	93.06	78.97	101.15	48.07
sla	50.66	23.52	74.42	0.00	125.34	88.73	70.17	98.08	109.14	74.52	101.95
ssd	97.18	109.11	77.22	125.34	0.00	78.39	75.12	110.86	79.86	89.01	48.69
sm	39.40	82.80	23.60	88.73	78.39	0.00	115.59	72.35	84.77	97.78	40.70
SRL	89.91	73.55	92.96	70.17	75.12	115.59	0.00	160.42	75.92	95.06	104.30
D	89.81	91.69	93.06	98.08	110.86	72.35	160.42	0.00	123.00	67.04	77.24
RTD	91.98	126.19	78.97	109.14	79.86	84.77	75.92	123.00	0.00	167.02	103.67
RN	90.74	55.07	101.15	74.52	89.01	97.78	95.06	67.04	167.02	0.00	71.92
Dr	63.00	83.21	48.07	101.95	48.69	40.70	104.30	77.24	103.67	71.92	0.00
N. of dimensions	4										
END	7.05										

UPFS and Lr											
	PC1	*PC2*	*PC3*	*PC4*	*PC5*	*PC6*	*PC7*	*PC8*	*PC9*	*PC10*	*PC11*
Eigenvalues	2.76	2.06	1.86	1.05	0.95	0.71	0.51	0.47	0.31	0.25	0.06
Cum. variance	25.05	43.82	60.73	70.24	78.90	85.31	89.98	94.29	97.14	99.43	100.00
Loadings	*PC1*	*PC2*	*PC3*	*PC4*	*PC5*	*PC6*	*PC7*	*PC8*	*PC9*	*PC10*	*PC11*
la	-0.79	0.10	-0.03	-0.06	0.10	-0.18	0.49	-0.03	0.25	0.08	0.00
ln	-0.49	-0.06	-0.60	-0.06	-0.41	0.29	-0.23	0.01	0.28	-0.11	0.04
ph	-0.79	0.23	0.29	-0.08	0.14	-0.03	0.00	0.28	-0.17	-0.32	0.04
sla	-0.48	0.05	-0.68	0.21	-0.20	0.13	0.19	-0.18	-0.36	0.04	-0.02
ssd	0.08	0.31	0.37	-0.70	-0.13	0.47	0.15	-0.09	-0.06	0.05	0.00
sm	-0.59	-0.07	0.42	-0.20	-0.24	-0.37	-0.25	-0.41	-0.04	0.01	0.00
SRL	0.07	0.75	-0.45	-0.18	0.37	-0.13	-0.13	-0.06	-0.01	0.10	0.15
D	-0.17	-0.91	0.25	0.09	0.06	0.17	0.07	-0.01	-0.05	0.09	0.17
RTD	0.16	0.51	0.34	0.24	-0.67	-0.14	0.09	0.22	-0.03	0.11	0.07
RN	-0.04	-0.49	-0.43	-0.57	-0.14	-0.32	-0.04	0.32	-0.09	0.12	-0.02
Lr	-0.78	0.15	0.25	0.17	0.19	0.22	-0.27	0.18	-0.01	0.29	-0.06
Angles	***la***	***ln***	***ph***	***sla***	***ssd***	***sm***	***SRL***	***D***	***RTD***	***N***	***Lr***
la	0.00	49.73	23.47	55.33	90.50	40.43	86.51	87.75	101.05	87.61	25.34
ln	49.73	0.00	72.86	20.61	110.99	84.76	72.82	91.70	128.22	59.66	71.19
ph	23.47	72.86	0.00	76.76	77.20	26.93	90.36	90.37	83.62	102.37	17.54
sla	55.33	20.61	76.76	0.00	125.26	94.33	68.89	98.24	113.26	77.05	70.15
ssd	90.50	110.99	77.20	125.26	0.00	69.49	75.50	108.80	77.61	83.10	93.81
sm	40.43	84.76	26.93	94.33	69.49	0.00	111.39	69.95	94.56	90.77	35.97
SRL	86.51	72.82	90.36	68.89	75.50	111.39	0.00	162.64	71.01	95.13	96.30
D	87.75	91.70	90.37	98.24	108.80	69.95	162.64	0.00	126.01	69.98	84.89
RTD	101.05	128.22	83.62	113.26	77.61	94.56	71.01	126.01	0.00	156.44	82.62
RN	87.61	59.66	102.37	77.05	83.10	90.77	95.13	69.98	156.44	0.00	109.74
Lr	25.34	71.19	17.54	70.15	93.81	35.97	96.30	84.89	82.62	109.74	0.00
N. of dimensions	4										
END	6.55										

Supplementary Table 4 Eigendecompositions of the **Herbaceous species** subset (N = 12,601) considering UPFS traits while progressively including root size traits. ***UPFS and Dr+Lr*** refers to eigendecomposition performed on UPFS traits along with both Lr and Dr; ***UPFS***, considers only UPFS traits; ***UPFS and Dr***, considers UPFS traits and maximum rooting depth; ***UPFS and Lr***, considers UPFS traits and maximum lateral root spread. *Cum. variance*, cumulative variance explained by each axis; *N. of dimensions*, number of relevant dimensions retained; *Angles*, angles across pairs of traits considering all relevant dimensions; *END*, effective number of dimensions.

Herbaceous

UPFS and Dr+Lr

	PC1	PC2	PC3	PC4	PC5	PC6	PC7	PC8	PC9	PC10	PC11	PC12
Eigenvalues	2.97	2.12	1.54	1.12	1.10	0.73	0.61	0.53	0.52	0.38	0.33	0.06
Cum. variance	24.78	42.42	55.22	64.54	73.70	79.81	84.92	89.34	93.64	96.78	99.51	100.00

Loadings	PC1	PC2	PC3	PC4	PC5	PC6	PC7	PC8	PC9	PC10	PC11	PC12
la	-0.64	-0.37	-0.27	0.14	0.08	0.23	-0.01	0.44	-0.14	-0.15	0.25	-0.02
ln	-0.02	-0.73	-0.10	-0.41	0.08	-0.28	-0.10	0.11	0.27	-0.25	-0.19	-0.03
ph	-0.62	-0.16	-0.43	0.08	-0.12	0.40	0.34	-0.11	0.09	0.02	-0.29	0.02
sla	0.22	-0.71	-0.09	0.06	0.43	0.14	0.03	-0.20	0.27	0.25	0.22	0.02
ssd	-0.07	0.66	-0.21	-0.39	-0.29	0.14	-0.02	0.14	0.45	0.06	0.16	-0.01
sm	-0.60	-0.24	0.07	-0.31	-0.25	0.23	-0.55	-0.18	-0.14	0.13	-0.03	0.00
SRL	0.63	0.00	-0.70	0.19	-0.09	0.03	-0.15	-0.07	-0.07	0.03	-0.03	-0.16
D	-0.57	-0.08	0.70	0.32	-0.11	-0.01	0.06	-0.02	0.19	0.04	-0.01	-0.15
RTD	-0.20	0.35	0.09	-0.57	0.65	0.12	0.13	0.00	-0.18	0.06	-0.05	-0.08
RN	0.22	-0.50	0.09	-0.43	-0.54	-0.09	0.36	0.03	-0.20	0.17	0.11	-0.03
Dr	-0.71	0.14	-0.31	-0.05	-0.02	-0.28	0.12	-0.43	-0.03	-0.21	0.22	-0.01
Lr	-0.67	0.10	-0.34	0.11	0.08	-0.49	-0.04	0.18	0.00	0.35	-0.09	0.01

Angles	la	ln	ph	sla	ssd	sm	SRL	D	RTD	N	Dr	Lr
la	0.00	67.71	24.60	74.12	109.20	50.12	104.55	71.80	93.95	96.73	40.94	35.14
ln	67.71	0.00	79.33	43.48	116.73	63.65	91.98	99.78	87.99	49.55	93.49	95.79
ph	24.60	79.33	0.00	92.74	87.58	49.94	94.80	81.94	97.32	95.74	27.21	25.32
sla	74.12	43.48	92.74	0.00	146.38	98.13	77.79	100.73	93.83	79.60	110.53	102.33
ssd	109.20	116.73	87.58	146.38	0.00	85.17	85.89	108.56	71.22	93.18	70.26	80.13
sm	50.12	63.65	49.94	98.13	85.17	0.00	128.97	62.65	86.08	67.06	49.32	59.64
SRL	104.55	91.98	94.80	77.79	85.89	128.97	0.00	146.46	112.25	87.22	108.40	102.79
D	71.80	99.78	81.94	100.73	108.56	62.65	146.46	0.00	96.80	97.01	77.32	77.64
RTD	93.95	87.99	97.32	93.83	71.22	86.08	112.25	96.80	0.00	111.69	76.71	80.26
RN	96.73	49.55	95.74	79.60	93.18	67.06	87.22	97.01	111.69	0.00	108.55	118.14
Dr	40.94	93.49	27.21	110.53	70.26	49.32	108.40	77.32	76.71	108.55	0.00	15.01
Lr	35.14	95.79	25.32	102.33	80.13	59.64	102.79	77.64	80.26	118.14	15.01	0.00

N. of dimensions	5
END	7.25

UPFS

	PC1	PC2	PC3	PC4	PC5	PC6	PC7	PC8	PC9	PC10
Eigenvalues	2.37	2.07	1.32	1.11	1.09	0.61	0.52	0.49	0.36	0.06
Cum. variance	23.67	44.36	57.58	68.69	79.60	85.71	90.87	95.78	99.40	100.00

Loadings	PC1	PC2	PC3	PC4	PC5	PC6	PC7	PC8	PC9	PC10
la	-0.67	0.19	-0.46	0.12	0.18	-0.05	-0.16	0.37	0.30	0.02
ln	-0.22	0.71	0.01	-0.43	-0.11	0.11	0.28	0.32	-0.25	0.03
ph	-0.53	0.03	-0.65	0.17	0.01	-0.35	0.09	-0.23	-0.27	-0.02
sla	-0.03	0.75	0.06	-0.10	0.43	-0.01	0.27	-0.32	0.26	-0.02
ssd	0.20	-0.61	-0.38	-0.23	-0.38	-0.01	0.44	0.03	0.22	0.01
sm	-0.67	0.01	-0.19	-0.17	-0.31	0.54	-0.14	-0.26	0.01	0.00
SRL	0.75	0.33	-0.46	0.23	0.02	0.16	-0.07	-0.05	-0.03	0.16
D	-0.72	-0.23	0.51	0.32	-0.01	-0.06	0.19	-0.04	0.01	0.15
RTD	-0.09	-0.41	-0.04	-0.77	0.40	-0.14	-0.18	-0.08	-0.02	0.09
RN	0.00	0.51	0.15	-0.20	-0.68	-0.36	-0.21	-0.10	0.14	0.03

Angles	la	ln	ph	sla	ssd	sm	SRL	D	RTD	N
la	0.00	73.89	22.32	74.46	103.25	46.66	103.42	73.29	91.46	99.07
ln	73.89	0.00	85.79	44.25	117.36	67.33	92.58	99.17	88.99	46.95
ph	22.32	85.79	0.00	90.98	83.94	47.90	93.59	82.96	94.33	99.72
sla	74.46	44.25	90.98	0.00	145.33	98.08	77.37	101.06	93.75	80.90
ssd	103.25	117.36	83.94	145.33	0.00	82.64	85.79	108.42	70.64	94.81
sm	46.66	67.33	47.90	98.08	82.64	0.00	126.91	63.99	84.89	71.29
SRL	103.42	92.58	93.59	77.37	85.79	126.91	0.00	146.49	111.93	87.34
D	73.29	99.17	82.96	101.06	108.42	63.99	146.49	0.00	96.89	96.93
RTD	91.46	88.99	94.33	93.75	70.64	84.89	111.93	96.89	0.00	112.60
RN	99.07	46.95	99.72	80.90	94.81	71.29	87.34	96.93	112.60	0.00

N. of dimensions	5
END	6.64

UPFS and Dr

	PC1	PC2	PC3	PC4	PC5	PC6	PC7	PC8	PC9	PC10	PC11
Eigenvalues	2.64	2.11	1.44	1.11	1.09	0.62	0.59	0.52	0.49	0.33	0.06
Cum. variance	24.01	43.17	56.29	66.39	76.31	81.92	87.31	92.01	96.46	99.46	100.00

Loadings	PC1	PC2	PC3	PC4	PC5	PC6	PC7	PC8	PC9	PC10	PC11
la	-0.64	0.33	-0.34	0.13	0.18	0.10	0.27	0.16	-0.40	0.22	-0.02
ln	-0.05	0.74	-0.06	-0.43	-0.11	0.01	-0.22	-0.26	-0.30	-0.23	-0.03
ph	-0.60	0.12	-0.55	0.18	0.02	-0.22	0.30	-0.10	0.23	-0.29	0.02
sla	0.17	0.73	-0.02	-0.11	0.43	0.01	0.06	-0.28	0.31	0.26	0.02
ssd	0.00	-0.66	-0.31	-0.23	-0.38	0.09	0.18	-0.45	-0.06	0.16	-0.01
sm	-0.65	0.18	-0.07	-0.16	-0.31	0.57	-0.11	0.13	0.26	-0.01	0.00
SRL	0.72	0.09	-0.60	0.23	0.02	0.13	-0.09	0.07	0.06	-0.02	-0.16
D	-0.65	0.00	0.64	0.31	-0.01	-0.05	0.03	-0.19	0.04	0.00	-0.15
RTD	-0.19	-0.37	0.02	-0.77	0.40	-0.08	0.14	0.18	0.08	-0.04	-0.08
RN	0.15	0.50	0.08	-0.21	-0.68	-0.32	0.19	0.20	0.10	0.14	-0.03
Dr	-0.64	-0.17	-0.38	-0.01	0.00	-0.32	-0.52	0.02	0.06	0.17	-0.01

Angles	la	ln	ph	sla	ssd	sm	SRL	D	RTD	N	Dr
la	0.00	71.43	24.20	73.16	107.72	48.22	103.92	72.40	92.86	98.28	41.11
ln	71.43	0.00	83.97	44.88	116.83	65.30	92.60	99.36	88.53	47.44	95.53
ph	24.20	83.97	0.00	91.16	86.45	49.68	93.56	83.00	95.37	99.35	27.64
sla	73.16	44.88	91.16	0.00	146.06	97.86	77.43	100.87	93.66	80.83	110.00
ssd	107.72	116.83	86.45	146.06	0.00	84.95	85.64	108.70	71.11	94.12	68.54
sm	48.22	65.30	49.68	97.86	84.95	0.00	127.96	63.16	85.74	69.85	44.79
SRL	103.92	92.60	93.56	77.43	85.64	127.96	0.00	146.44	111.95	87.35	109.71
D	72.40	99.36	83.00	100.87	108.70	63.16	146.44	0.00	96.91	96.88	76.68
RTD	92.86	88.53	95.37	93.66	71.11	85.74	111.95	96.91	0.00	112.41	75.51
RN	98.28	47.44	99.35	80.83	94.12	69.85	87.35	96.88	112.41	0.00	107.79
Dr	41.11	95.53	27.64	110.00	68.54	44.79	109.71	76.68	75.51	107.79	0.00

N. of dimensions	5
END	7.00

UPFS and Lr

	PC1	PC2	PC3	PC4	PC5	PC6	PC7	PC8	PC9	PC10	PC11
Eigenvalues	2.59	2.09	1.45	1.12	1.10	0.70	0.61	0.52	0.43	0.35	0.06
Cum. variance	23.50	42.54	55.71	65.85	75.84	82.17	87.70	92.40	96.28	99.45	100.00

Loadings	PC1	PC2	PC3	PC4	PC5	PC6	PC7	PC8	PC9	PC10	PC11
la	-0.68	0.29	-0.39	0.11	0.07	-0.08	0.06	-0.18	0.45	0.18	0.02
ln	-0.10	0.73	-0.03	-0.41	0.07	0.31	-0.05	0.26	0.16	-0.29	0.03
ph	-0.58	0.10	-0.52	0.06	-0.13	-0.40	0.29	0.10	-0.20	-0.24	-0.02
sla	0.12	0.74	-0.02	0.05	0.44	-0.20	-0.03	0.29	-0.17	0.30	-0.02
ssd	0.05	-0.65	-0.27	-0.39	-0.32	-0.05	0.01	0.44	0.13	0.18	0.01
sm	-0.64	0.15	-0.01	-0.31	-0.27	-0.15	-0.58	-0.13	-0.15	0.04	0.00
SRL	0.71	0.14	-0.61	0.19	-0.08	-0.02	-0.16	-0.07	-0.06	-0.01	0.16
D	-0.67	-0.06	0.60	0.34	-0.08	-0.02	0.05	0.19	-0.03	0.01	0.15
RTD	-0.15	-0.38	0.04	-0.61	0.62	-0.11	0.12	-0.18	-0.07	-0.01	0.08
RN	0.15	0.52	0.19	-0.39	-0.55	0.03	0.36	-0.20	-0.11	0.18	0.03
Lr	-0.58	-0.16	-0.43	0.09	0.08	0.59	0.08	-0.01	-0.26	0.13	-0.01

Angles	la	ln	ph	sla	ssd	sm	SRL	D	RTD	N	Lr
la	0.00	69.67	22.54	76.24	104.53	48.80	103.68	73.18	92.55	98.08	32.41
ln	69.67	0.00	80.61	42.88	116.98	64.82	92.08	99.51	88.24	49.45	97.06
ph	22.54	80.61	0.00	93.20	85.08	48.64	94.43	82.37	96.27	96.88	25.48
sla	76.24	42.88	93.20	0.00	145.98	98.64	77.78	100.86	94.02	79.23	102.10
ssd	104.53	116.98	85.08	145.98	0.00	83.27	85.85	108.47	70.84	94.16	79.28
sm	48.80	64.82	48.64	98.64	83.27	0.00	127.91	63.51	85.44	68.69	58.36
SRL	103.68	92.08	94.43	77.78	85.85	127.91	0.00	146.52	112.14	87.31	103.28
D	73.18	99.51	82.37	100.86	108.47	63.51	146.52	0.00	96.85	96.82	76.57
RTD	92.55	88.24	96.27	94.02	70.84	85.44	112.14	96.85	0.00	111.94	79.83
RN	98.08	49.45	96.88	79.23	94.16	68.69	87.31	96.82	111.94	0.00	119.70
Lr	32.41	97.06	25.48	102.10	79.28	58.36	103.28	76.57	79.83	119.70	0.00

N. of dimensions	5
END	7.10

Supplementary Table 5 Eigendecompositions of the GSPFF traits subset. ***GSPFF and Dr+Lr*** refers to eigendecomposition performed on GSPFF traits along with both Lr and Dr; ***GSPFF***, considers only GSPFF traits; ***GSPFF and Dr***, considers GSPFF traits and maximum rooting depth; ***GSPFF and Lr***, considers GSPFF traits and maximum lateral spread. *Cum. variance,* cumulative variance explained by each axis; *N. of dimensions*, number of relevant dimensions retained; *Angles*, angles across pairs of traits considering all relevant dimensions; *END*, effective number of dimensions.

GSPFF and Dr+Lr								
	PC1	*PC2*	*PC3*	*PC4*	*PC5*	*PC6*	*PC7*	*PC8*
Eigenvalues	3.76	1.68	0.77	0.53	0.47	0.40	0.25	0.13
Cum. variance	47.01	68.04	77.67	84.30	90.22	95.24	98.37	100.00
Loadings	*PC1*	*PC2*	*PC3*	*PC4*	*PC5*	*PC6*	*PC7*	*PC8*
la	-0.46	-0.62	0.53	-0.17	-0.19	0.16	-0.16	-0.08
ln	0.22	-0.78	-0.39	0.28	0.07	0.30	-0.06	0.04
ph	-0.89	-0.23	0.12	-0.16	0.16	0.01	0.14	0.27
sla	0.48	-0.75	-0.07	-0.20	0.08	-0.31	0.23	-0.09
ssd	-0.87	0.15	0.03	0.10	0.22	0.23	0.26	-0.19
sm	-0.75	-0.19	0.12	0.52	-0.10	-0.33	-0.02	-0.01
Dr	-0.72	0.00	-0.45	-0.17	-0.50	0.01	0.08	-0.01
Lr	-0.81	-0.04	-0.32	-0.21	0.30	-0.16	-0.28	-0.07
Angles	***la***	***ln***	***ph***	***sla***	***ssd***	***sm***	***Dr***	***Lr***
la	0.00	51.84	39.13	68.58	63.72	39.53	54.11	50.59
ln	51.84	0.00	90.97	16.74	115.56	91.37	105.95	102.43
ph	39.13	90.97	0.00	107.71	24.59	0.41	14.98	11.47
sla	68.58	16.74	107.71	0.00	132.30	108.11	122.69	119.17
ssd	63.72	115.56	24.59	132.30	0.00	24.19	9.61	13.13
sm	39.53	91.37	0.41	108.11	24.19	0.00	14.57	11.06
Dr	54.11	105.95	14.98	122.69	9.61	14.57	0.00	3.51
Lr	50.59	102.43	11.47	119.17	13.13	11.06	3.51	0.00
N. of dimensions	2							
END	3.49							

GSPFF						
	PC1	*PC2*	*PC3*	*PC4*	*PC5*	*PC6*
Eigenvalues	2.78	1.68	0.61	0.43	0.35	0.14
Cum. variance	46.38	74.39	84.53	91.77	97.59	100.00
Loadings	*PC1*	*PC2*	*PC3*	*PC4*	*PC5*	*PC6*
la	-0.52	0.64	-0.48	0.01	0.30	-0.07
ln	0.28	0.77	0.49	-0.19	0.20	0.05
ph	-0.88	0.25	-0.08	-0.20	-0.23	0.25
sla	0.51	0.74	-0.12	0.06	-0.40	-0.12
ssd	-0.89	-0.13	0.19	-0.29	-0.09	-0.25
sm	-0.78	0.21	0.28	0.52	-0.03	-0.01
Angles	***la***	***ln***	***ph***	***sla***	***ssd***	***sm***
la	0.00	58.76	35.21	73.77	59.62	35.99
ln	58.76	0.00	93.97	15.01	118.38	94.75
ph	35.21	93.97	0.00	108.98	24.41	0.78
sla	73.77	15.01	108.98	0.00	133.39	109.76
ssd	59.62	118.38	24.41	133.39	0.00	23.63
sm	35.99	94.75	0.78	109.76	23.63	0.00
N. of dimensions	2					
END	3.19					

GSPFF and Dr							
	PC1	*PC2*	*PC3*	*PC4*	*PC5*	*PC6*	*PC7*
Eigenvalues	3.19	1.68	0.71	0.52	0.42	0.35	0.14
Cum. variance	45.51	69.52	79.64	87.02	93.04	97.99	100.00
Loadings	*PC1*	*PC2*	*PC3*	*PC4*	*PC5*	*PC6*	*PC7*
la	-0.49	0.64	-0.46	0.21	0.02	0.30	0.07
ln	0.25	0.78	0.43	-0.23	-0.25	0.18	-0.05
ph	-0.88	0.25	-0.12	0.08	-0.17	-0.24	-0.25
sla	0.51	0.74	-0.02	0.13	0.12	-0.39	0.12
ssd	-0.88	-0.14	0.03	-0.14	-0.34	-0.12	0.23
sm	-0.77	0.21	0.03	-0.45	0.41	-0.02	0.01
Dr	-0.70	0.01	0.54	0.42	0.18	0.05	0.04
Angles	***la***	***ln***	***ph***	***sla***	***ssd***	***sm***	***Dr***
la	0.00	55.25	36.88	71.84	61.47	37.54	52.18
ln	55.25	0.00	92.13	16.59	116.72	92.79	107.43
ph	36.88	92.13	0.00	108.72	24.59	0.66	15.30
sla	71.84	16.59	108.72	0.00	133.31	109.38	124.02
ssd	61.47	116.72	24.59	133.31	0.00	23.93	9.29
sm	37.54	92.79	0.66	109.38	23.93	0.00	14.65
Dr	52.18	107.43	15.30	124.02	9.29	14.65	0.00
N. of dimensions	2						
END	3.48						

GSPFF and Lr							
	PC1	*PC2*	*PC3*	*PC4*	*PC5*	*PC6*	*PC7*
Eigenvalues	3.32	1.68	0.68	0.52	0.40	0.26	0.13
Cum. variance	47.42	71.46	81.19	88.69	94.42	98.13	100.00
Loadings	*PC1*	*PC2*	*PC3*	*PC4*	*PC5*	*PC6*	*PC7*
la	-0.48	0.62	0.56	0.08	-0.16	-0.17	-0.09
ln	0.24	0.78	-0.43	-0.22	-0.30	-0.06	0.04
ph	-0.90	0.23	0.04	0.20	-0.01	0.15	0.27
sla	0.48	0.75	-0.03	0.22	0.31	0.23	-0.09
ssd	-0.89	-0.16	-0.11	-0.02	-0.24	0.29	-0.19
sm	-0.76	0.19	0.01	-0.53	0.33	-0.02	-0.01
Lr	-0.79	0.04	-0.41	0.32	0.15	-0.26	-0.08
Angles	***la***	***ln***	***ph***	***sla***	***ssd***	***sm***	***Lr***
la	0.00	54.71	37.81	70.27	62.06	38.27	49.00
ln	54.71	0.00	92.52	15.56	116.77	92.98	103.71
ph	37.81	92.52	0.00	108.09	24.25	0.45	11.19
sla	70.27	15.56	108.09	0.00	132.34	108.54	119.27
ssd	62.06	116.77	24.25	132.34	0.00	23.80	13.07
sm	38.27	92.98	0.45	108.54	23.80	0.00	10.73
Lr	49.00	103.71	11.19	119.27	13.07	10.73	0.00
N. of dimensions	2						
END	3.30						

Supplementary Table 6 Eigendecompositions of the RES traits. ***RES and Dr+Lr*** refers to eigendecomposition performed on RES traits along with both Lr and Dr; ***RES***, considers only RES traits; ***RES and Dr***, considers RES traits and maximum rooting depth; ***RES and Lr***, considers RES traits and maximum lateral root spread. *Cum. variance,* cumulative variance explained by each axis; *N. of dimensions*, number of relevant dimensions retained; *Angles*, angles across pairs of traits considering all relevant dimensions; *END*, effective number of dimensions.

RES and Dr+Lr						
	PC1	*PC2*	*PC3*	*PC4*	*PC5*	*PC6*
Eigenvalues	2.01	1.55	1.15	0.78	0.40	0.10
Cum. variance	33.54	59.45	78.68	91.72	98.41	100.00
Loadings	*PC1*	*PC2*	*PC3*	*PC4*	*PC5*	*PC6*
SRL	0.84	-0.21	0.43	0.16	-0.06	-0.20
D	-0.79	0.51	-0.19	0.19	0.01	-0.21
RTD	0.05	-0.61	-0.61	-0.49	0.06	-0.11
RN	-0.01	0.56	0.46	-0.69	0.05	-0.02
Dr	-0.62	-0.51	0.36	-0.10	-0.45	-0.01
Lr	-0.54	-0.55	0.47	0.05	0.43	-0.01
Angles	***SRL***	***D***	***RTD***	***N***	***Dr***	***Lr***
SRL	0.00	156.98	96.27	84.02	107.55	99.00
D	156.98	0.00	106.38	73.05	78.98	86.04
RTD	96.27	106.38	0.00	174.59	85.06	88.02
RN	84.02	73.05	174.59	0.00	100.02	97.28
Dr	107.55	78.98	85.06	100.02	0.00	8.81
Lr	99.00	86.04	88.02	97.28	8.81	0.00
N. of dimensions	3					
END	4.20					

RES				
	PC1	*PC2*	*PC3*	*PC4*
Eigenvalues	1.82	1.31	0.78	0.10
Cum. variance	45.49	78.13	97.56	100.00
Loadings	*PC1*	*PC2*	*PC3*	*PC4*
SRL	0.88	0.40	-0.17	-0.20
D	-0.96	-0.03	-0.17	-0.21
RTD	0.27	-0.81	0.51	-0.11
RN	-0.22	0.70	0.68	-0.02
Angles	***SRL***	***D***	***RTD***	***N***
SRL	0.00	157.42	95.80	83.39
D	157.42	0.00	106.77	74.03
RTD	95.80	106.77	0.00	179.19
RN	83.39	74.03	179.19	0.00
N. of dimensions	2			
END	2.84			

RES and Dr					
	PC1	*PC2*	*PC3*	*PC4*	*PC5*
Eigenvalues	1.89	1.38	0.87	0.77	0.10
Cum. variance	37.73	65.31	82.62	98.08	100.00
Loadings	*PC1*	*PC2*	*PC3*	*PC4*	*PC5*
SRL	0.91	-0.24	0.19	-0.21	-0.20
D	-0.93	-0.14	-0.22	-0.14	-0.21
RTD	0.18	0.81	-0.11	0.53	-0.11
RN	-0.15	-0.69	0.33	0.63	-0.02
Dr	-0.38	0.41	0.81	-0.17	-0.01
Angles	***SRL***	***D***	***RTD***	***N***	***Dr***
SRL	0.00	156.55	92.77	87.13	147.25
D	156.55	0.00	110.68	69.42	56.20
RTD	92.77	110.68	0.00	179.90	54.48
RN	87.13	69.42	179.90	0.00	125.62
Dr	147.25	56.20	54.48	125.62	0.00
N. of dimensions	2				
END	3.67				

RES and Lr					
	PC1	*PC2*	*PC3*	*PC4*	*PC5*
Eigenvalues	1.84	1.35	0.95	0.77	0.10
Cum. variance	36.80	63.85	82.78	98.09	100.00
Loadings	*PC1*	*PC2*	*PC3*	*PC4*	*PC5*
SRL	0.89	-0.31	0.21	-0.14	0.20
D	-0.95	-0.05	-0.08	-0.20	0.21
RTD	0.24	0.77	-0.36	0.45	0.11
RN	-0.18	-0.71	-0.01	0.68	0.02
Lr	-0.21	0.38	0.87	0.21	0.02
Angles	***SRL***	***D***	***RTD***	***N***	***Dr***
SRL	0.00	157.63	92.25	84.91	138.59
D	157.63	0.00	110.12	72.72	63.78
RTD	92.25	110.12	0.00	177.16	46.34
RN	84.91	72.72	177.16	0.00	136.50
Lr	138.59	63.78	46.34	136.50	0.00
N. of dimensions	2				
END	3.73				

Supplementary Table 7 Principal component analysis (PCA) based on species with complete trait observations, performed on UPFS traits subsets. ***UPFS and Dr+Lr*** refers to PCA performed on UPFS traits along with both Lr and Dr; ***UPFS***, refers to PCA on UPFS traits; ***UPFS and Dr***, refers to PCA on UPFS traits and maximum rooting depth; ***UPFS and Lr***, refers to PCA on UPFS traits and maximum lateral spread. *Cum. variance,* cumulative variance explained by each axis; *N. of dimensions*, number of relevant dimensions retained; *Angles*, angles across pairs of traits considering all relevant dimensions; *END*, effective number of dimensions; *N. of species*, number of species on which PCA was performed (i.e., species with complete traits observations).

UPFS and Dr+Lr												
	PC1	*PC2*	*PC3*	*PC4*	*PC5*	*PC6*	*PC7*	*PC8*	*PC9*	*PC10*	*PC11*	*PC12*
Eigenvalues	4.16	1.95	1.80	1.22	0.89	0.59	0.35	0.30	0.28	0.16	0.13	0.09
Cum. variance	34.94	51.28	66.41	76.63	84.13	89.07	92.04	94.53	96.91	98.22	99.28	100.00
Loadings	*PC1*	*PC2*	*PC3*	*PC4*	*PC5*	*PC6*	*PC7*	*PC8*	*PC9*	*PC10*	*PC11*	*PC12*
la	0.35	0.50	0.36	0.58	0.14	0.03	0.08	0.24	0.25	0.07	0.02	0.02
ln	-0.34	0.53	0.60	-0.04	-0.26	0.21	0.12	-0.30	-0.03	0.14	0.01	-0.02
ph	0.88	-0.05	0.33	-0.10	0.05	-0.02	-0.08	-0.11	0.14	-0.11	-0.07	-0.20
sla	-0.57	0.27	0.48	0.38	-0.04	-0.20	-0.35	0.04	-0.22	-0.08	0.01	-0.02
ssd	0.82	-0.29	0.14	-0.19	-0.12	-0.19	-0.18	0.08	-0.06	0.28	0.08	-0.02
sm	0.72	0.30	0.18	-0.04	-0.08	-0.45	0.32	-0.01	-0.19	-0.08	0.01	0.03
SRL	-0.24	-0.71	0.57	0.06	0.09	0.10	0.15	0.03	-0.01	-0.07	0.23	-0.03
D	0.03	0.77	-0.35	-0.33	0.33	0.00	-0.08	-0.03	0.01	-0.03	0.21	-0.04
RTD	0.31	0.04	-0.49	0.37	-0.70	0.03	-0.04	-0.04	0.06	-0.07	0.12	-0.03
RN	-0.34	0.22	0.37	-0.65	-0.41	0.03	-0.02	0.28	0.09	-0.07	-0.02	0.02
Dr	0.78	0.14	-0.02	0.05	0.01	0.49	0.03	0.16	-0.30	-0.02	-0.02	-0.02
Lr	0.87	-0.02	0.30	-0.09	0.04	0.10	-0.20	-0.16	0.09	-0.11	0.02	0.19
Angles	*la*	*ln*	*ph*	*sla*	*ssd*	*sm*	*SRL*	*D*	*RTD*	*N*	*Dr*	*Lr*
la	0.00	64.88	67.05	66.01	84.65	52.71	103.42	84.43	74.82	108.96	60.19	65.41
ln	64.88	0.00	98.75	37.16	115.61	88.21	86.47	75.60	131.36	49.51	107.43	98.48
ph	67.05	98.75	0.00	118.56	19.97	25.68	89.68	95.85	83.70	98.88	27.44	2.48
sla	66.01	37.16	118.56	0.00	133.81	111.50	72.67	97.88	115.80	75.63	125.04	118.43
ssd	84.65	115.61	19.97	133.81	0.00	42.12	84.45	102.96	80.66	102.59	35.35	21.44
sm	52.71	88.21	25.68	111.50	42.12	0.00	111.77	74.00	76.25	97.65	19.98	23.39
SRL	103.42	86.47	89.68	72.67	84.45	111.77	0.00	154.34	123.54	83.06	113.39	91.86
D	84.43	75.60	95.85	97.88	102.96	74.00	154.34	0.00	81.98	71.41	79.80	94.09
RTD	74.82	131.36	83.70	115.80	80.66	76.25	123.54	81.98	0.00	152.21	59.79	82.13
RN	108.96	49.51	98.88	75.63	102.59	97.65	83.06	71.41	152.21	0.00	114.00	99.74
Dr	60.19	107.43	27.44	125.04	35.35	19.98	113.39	79.80	59.79	114.00	0.00	25.26
Lr	65.41	98.48	2.48	118.43	21.44	23.39	91.86	94.09	82.13	99.74	25.26	0.00
N. of dimensions	4								***N. of species***			134
END	5.19											

UPFS										
	PC1	*PC2*	*PC3*	*PC4*	*PC5*	*PC6*	*PC7*	*PC8*	*PC9*	*PC10*
Eigenvalues	2.91	2.01	1.62	1.12	0.89	0.48	0.34	0.31	0.19	0.08
Cum. variance	29.24	49.40	65.65	76.92	85.89	90.74	94.11	97.24	99.16	100.00
Loadings	*PC1*	*PC2*	*PC3*	*PC4*	*PC5*	*PC6*	*PC7*	*PC8*	*PC9*	*PC10*
la	0.21	0.44	0.59	0.23	0.37	0.44	0.04	0.12	0.04	0.00
ln	-0.58	0.48	0.47	0.04	-0.12	-0.08	-0.33	-0.27	0.10	0.00
ph	0.84	0.12	0.32	-0.17	0.02	-0.03	-0.04	-0.20	-0.32	-0.02
sla	-0.62	0.25	0.50	0.24	0.06	-0.30	0.37	-0.02	-0.07	-0.01
ssd	0.83	-0.12	0.24	-0.23	-0.12	-0.02	0.22	-0.22	0.26	0.00
sm	0.69	0.39	0.30	0.02	-0.16	-0.34	-0.14	0.33	0.06	0.00
SRL	-0.24	-0.73	0.50	-0.30	0.13	-0.02	-0.08	0.09	0.01	-0.19
D	0.08	0.81	-0.52	-0.09	0.11	-0.01	0.05	-0.04	0.02	-0.20
RTD	0.26	-0.25	0.03	0.77	-0.50	0.11	-0.01	-0.03	-0.01	-0.10
RN	-0.36	0.30	0.18	-0.49	-0.65	0.24	0.11	0.11	-0.05	0.00
Angles	*la*	*ln*	*ph*	*sla*	*ssd*	*sm*	*SRL*	*D*	*RTD*	*N*
la	0.00	58.17	59.06	62.02	72.71	42.40	101.05	86.56	78.58	84.55
ln	58.17	0.00	110.84	20.23	122.96	95.43	89.23	83.97	107.72	48.05
ph	59.06	110.84	0.00	118.18	16.02	23.22	95.27	89.02	84.64	101.20
sla	62.02	20.23	118.18	0.00	128.00	103.94	80.21	98.93	92.18	63.90
ssd	72.71	122.96	16.02	128.00	0.00	38.92	85.15	98.61	83.95	106.23
sm	42.40	95.43	23.22	103.94	38.92	0.00	112.04	74.91	81.53	97.96
SRL	101.05	89.23	95.27	80.21	85.15	112.04	0.00	154.42	96.87	81.26
D	86.56	83.97	89.02	98.93	98.61	74.91	154.42	0.00	108.60	76.05
RTD	78.58	107.72	84.64	92.18	83.95	81.53	96.87	108.60	0.00	155.38
RN	84.55	48.05	101.20	63.90	106.23	97.96	81.26	76.05	155.38	0.00
N. of dimensions	4							***N. of species***		264
END	5.61									

UPFS and Dr											
	PC1	*PC2*	*PC3*	*PC4*	*PC5*	*PC6*	*PC7*	*PC8*	*PC9*	*PC10*	*PC11*
Eigenvalues	3.60	1.87	1.61	1.21	0.95	0.55	0.32	0.30	0.25	0.15	0.12
Cum. variance	32.97	50.07	64.76	75.88	84.60	89.62	92.54	95.25	97.51	98.93	100.00
Loadings	*PC1*	*PC2*	*PC3*	*PC4*	*PC5*	*PC6*	*PC7*	*PC8*	*PC9*	*PC10*	*PC11*
la	0.21	0.49	0.58	0.41	0.28	0.03	0.28	0.16	0.14	0.05	0.00
ln	-0.49	0.57	0.47	-0.10	-0.18	0.20	-0.18	0.12	-0.27	0.09	0.02
ph	0.84	-0.01	0.29	-0.25	0.06	-0.03	0.13	0.03	-0.22	-0.26	-0.01
sla	-0.67	0.28	0.44	0.21	0.06	-0.27	-0.03	-0.38	-0.02	-0.08	-0.03
ssd	0.81	-0.21	0.24	-0.23	-0.14	-0.20	0.14	-0.15	-0.12	0.26	0.00
sm	0.72	0.35	0.24	-0.15	-0.08	-0.33	-0.33	0.12	0.18	-0.02	-0.02
SRL	-0.37	-0.67	0.54	-0.19	0.10	0.07	-0.05	0.08	0.05	0.01	-0.23
D	0.14	0.70	-0.55	-0.20	0.28	0.00	0.05	-0.04	-0.06	0.04	-0.21
RTD	0.33	0.00	-0.12	0.66	-0.64	-0.02	0.03	0.03	-0.05	-0.03	-0.13
RN	-0.38	0.33	0.09	-0.59	-0.55	0.07	0.21	-0.02	0.18	-0.04	-0.01
Dr	0.77	0.09	0.18	0.04	0.02	0.52	-0.13	-0.25	0.12	0.00	-0.01
Angles	*la*	*ln*	*ph*	*sla*	*ssd*	*sm*	*SRL*	*D*	*RTD*	*N*	*Dr*
la	0.00	58.79	73.01	63.79	82.15	58.20	101.24	91.96	66.30	98.68	62.36
ln	58.79	0.00	108.14	29.72	118.53	92.26	85.24	83.59	115.16	47.05	110.19
ph	73.01	108.14	0.00	126.98	13.54	24.91	96.54	89.75	83.45	101.72	20.90
sla	63.79	29.72	126.98	0.00	133.47	114.52	72.26	102.75	102.10	67.15	124.84
ssd	82.15	118.53	13.54	133.47	0.00	38.12	88.65	98.46	82.72	107.94	27.71
sm	58.20	92.26	24.91	114.52	38.12	0.00	114.78	71.85	80.10	94.10	22.88
SRL	101.24	85.24	96.54	72.26	88.65	114.78	0.00	152.48	115.69	83.73	109.60
D	91.96	83.59	89.75	102.75	98.46	71.85	152.48	0.00	91.54	70.42	84.88
RTD	66.30	115.16	83.45	102.10	82.72	80.10	115.69	91.54	0.00	154.55	63.94
RN	98.68	47.05	101.72	67.15	107.94	94.10	83.73	70.42	154.55	0.00	115.79
Dr	62.36	110.19	20.90	124.84	27.71	22.88	109.60	84.88	63.94	115.79	0.00
N. of dimensions	4								***N. of species***		158
END	5.42										

UPFS and Lr											
	PC1	*PC2*	*PC3*	*PC4*	*PC5*	*PC6*	*PC7*	*PC8*	*PC9*	*PC10*	*PC11*
Eigenvalues	3.64	1.92	1.80	1.21	0.89	0.44	0.35	0.29	0.15	0.13	0.09
Cum. variance	33.35	50.94	67.43	78.53	86.71	90.71	93.94	96.61	98.03	99.19	100.00
Loadings	*PC1*	*PC2*	*PC3*	*PC4*	*PC5*	*PC6*	*PC7*	*PC8*	*PC9*	*PC10*	*PC11*
la	0.32	0.51	0.36	0.58	0.14	0.12	0.09	0.33	0.06	0.01	0.01
ln	-0.38	0.50	0.60	-0.05	-0.27	0.23	0.13	-0.27	0.14	0.00	-0.02
ph	0.89	0.00	0.31	-0.10	0.04	0.13	-0.07	-0.03	-0.14	-0.09	-0.21
sla	-0.57	0.24	0.49	0.38	-0.03	-0.29	-0.36	-0.08	-0.07	0.02	-0.02
ssd	0.85	-0.24	0.14	-0.18	-0.11	-0.16	-0.19	0.04	0.28	0.07	-0.03
sm	0.74	0.34	0.17	-0.02	-0.08	-0.43	0.30	-0.10	-0.07	0.01	0.04
SRL	-0.20	-0.73	0.58	0.04	0.09	0.03	0.14	0.02	-0.06	0.23	-0.03
D	-0.01	0.78	-0.35	-0.32	0.33	0.03	-0.08	-0.02	-0.02	0.21	-0.05
RTD	0.29	0.05	-0.49	0.38	-0.70	0.08	-0.03	-0.01	-0.07	0.12	-0.03
RN	-0.35	0.21	0.37	-0.65	-0.41	-0.04	-0.03	0.29	-0.07	-0.01	0.02
Lr	0.87	0.03	0.29	-0.08	0.03	0.22	-0.18	-0.09	-0.10	0.03	0.19
Angles	*la*	*ln*	*ph*	*sla*	*ssd*	*sm*	*SRL*	*D*	*RTD*	*N*	*Lr*
la	0.00	66.20	66.59	65.04	83.28	52.13	103.64	84.54	75.16	109.14	65.21
ln	66.20	0.00	100.22	36.26	116.52	90.36	85.93	75.77	131.94	48.89	99.93
ph	66.59	100.22	0.00	117.71	19.15	25.51	90.34	95.59	84.11	99.53	1.96
sla	65.04	36.26	117.71	0.00	132.63	110.63	72.97	97.74	114.97	75.94	117.46
ssd	83.28	116.52	19.15	132.63	0.00	41.05	85.43	102.47	80.72	103.51	20.37
sm	52.13	90.36	25.51	110.63	41.05	0.00	112.39	74.18	75.92	99.08	23.65
SRL	103.64	85.93	90.34	72.97	85.43	112.39	0.00	154.00	123.84	82.79	92.02
D	84.54	75.77	95.59	97.74	102.47	74.18	154.00	0.00	81.94	71.33	94.23
RTD	75.16	131.94	84.11	114.97	80.72	75.92	123.84	81.94	0.00	152.04	82.92
RN	109.14	48.89	99.53	75.94	103.51	99.08	82.79	71.33	152.04	0.00	100.18
Lr	65.21	99.93	1.96	117.46	20.37	23.65	92.02	94.23	82.92	100.18	0.00
N. of dimensions	4								***N. of species***		135
END	5.20										

Supplementary Table 8 Principal component analysis (PCA) based on species with complete trait observations, performed on GSPFF traits. ***GSPFF and Dr+Lr*** refers to PCA performed on GSPFF traits along with both Lr and Dr; ***GSPFF***, refers to PCA on GSPFF traits; ***GSPFF and Dr***, refers to PCA on GSPFF traits and maximum rooting depth; ***GSPFF and Lr***, refers to PCA on GSPFF traits and maximum lateral spread. *Cum. variance,* cumulative variance explained by each axis; *N. of dimensions*, number of relevant dimensions retained; *Angles*, angles across pairs of traits considering all relevant dimensions; *END*, effective number of dimensions; *N. of species*, number of species on which PCA was performed (i.e., species with complete traits observations).

GSPFF and Dr+Lr								
	PC1	*PC2*	*PC3*	*PC4*	*PC5*	*PC6*	*PC7*	*PC8*
Eigenvalues	3.72	1.75	0.67	0.61	0.50	0.36	0.22	0.16
Cum. variance	46.58	68.55	76.89	84.48	90.72	95.22	98.00	100.00
Loadings	*PC1*	*PC2*	*PC3*	*PC4*	*PC5*	*PC6*	*PC7*	*PC8*
la	0.28	0.73	0.55	0.19	0.11	0.08	0.16	0.03
ln	-0.26	0.77	-0.35	-0.38	-0.07	0.26	0.09	-0.01
ph	0.89	0.18	-0.12	0.15	0.16	0.11	-0.13	-0.29
sla	-0.51	0.61	-0.34	0.33	0.17	-0.33	-0.03	0.03
ssd	0.80	-0.30	-0.31	0.18	0.00	0.01	0.35	0.03
sm	0.71	0.33	-0.01	0.11	-0.59	-0.11	-0.08	0.03
Dr	0.76	0.13	0.08	-0.50	0.15	-0.36	0.04	-0.02
Lr	0.89	0.08	-0.12	0.04	0.22	0.15	-0.19	0.26
Angles	*la*	*ln*	*ph*	*sla*	*ssd*	*sm*	*Dr*	*Lr*
la	0.00	39.90	57.63	60.75	89.71	44.48	59.58	64.23
ln	39.90	0.00	97.53	20.85	129.60	84.38	99.48	104.13
ph	57.63	97.53	0.00	118.38	32.07	13.15	1.95	6.60
sla	60.75	20.85	118.38	0.00	150.45	105.23	120.33	124.98
ssd	89.71	129.60	32.07	150.45	0.00	45.23	30.12	25.48
sm	44.48	84.38	13.15	105.23	45.23	0.00	15.10	19.75
Dr	59.58	99.48	1.95	120.33	30.12	15.10	0.00	4.65
Lr	64.23	104.13	6.60	124.98	25.48	19.75	4.65	0.00
**N. of dimensions**	2					**N. of species**		494
**END**	3.51							

GSPFF						
	PC1	PC2	PC3	PC4	PC5	PC6
Eigenvalues	2.87	1.60	0.56	0.40	0.37	0.19
Cum. variance	47.83	74.49	83.85	90.58	96.82	100.00
Loadings	*PC1*	*PC2*	*PC3*	*PC4*	*PC5*	*PC6*
la	0.37	0.75	0.50	0.19	0.07	0.10
ln	-0.43	0.71	-0.48	0.26	0.12	0.00
ph	0.88	0.25	-0.05	-0.12	0.21	-0.32
sla	-0.62	0.60	0.03	-0.51	-0.01	0.03
ssd	0.88	-0.09	-0.22	-0.16	0.26	0.28
sm	0.78	0.33	-0.18	-0.03	-0.50	0.02
Angles	*la*	*ln*	*ph*	*sla*	*ssd*	*sm*
la	0.00	57.51	48.06	72.15	69.33	40.86
ln	57.51	0.00	105.56	14.65	126.84	98.37
ph	48.06	105.56	0.00	120.21	21.28	7.19
sla	72.15	14.65	120.21	0.00	141.49	113.02
ssd	69.33	126.84	21.28	141.49	0.00	28.47
sm	40.86	98.37	7.19	113.02	28.47	0.00
N. of dimensions	2			***N. of species***		1734
END	3.14					

GSPFF and Dr							
	PC1	*PC2*	*PC3*	*PC4*	*PC5*	*PC6*	*PC7*
Eigenvalues	3.24	1.56	0.65	0.53	0.45	0.35	0.21
Cum. variance	46.38	68.69	77.96	85.59	91.97	96.97	100.00
Loadings	*PC1*	*PC2*	*PC3*	*PC4*	*PC5*	*PC6*	*PC7*
la	0.18	0.79	0.50	0.27	0.04	0.09	0.09
ln	-0.46	0.68	-0.45	-0.07	-0.18	0.29	0.01
ph	0.86	0.21	0.03	-0.18	0.22	0.16	-0.33
sla	-0.67	0.48	-0.03	-0.33	0.37	-0.26	0.04
ssd	0.86	-0.12	-0.05	-0.29	0.15	0.19	0.31
sm	0.73	0.38	0.01	-0.25	-0.41	-0.29	0.00
Dr	0.72	0.21	-0.44	0.41	0.19	-0.20	0.04
Angles	*la*	*ln*	*ph*	*sla*	*ssd*	*sm*	*Dr*
la	0.00	47.10	63.77	67.03	85.41	50.28	61.31
ln	47.10	0.00	110.87	19.93	132.51	97.38	108.41
ph	63.77	110.87	0.00	130.79	21.65	13.48	2.46
sla	67.03	19.93	130.79	0.00	152.44	117.31	128.33
ssd	85.41	132.51	21.65	152.44	0.00	35.13	24.11
sm	50.28	97.38	13.48	117.31	35.13	0.00	11.02
Dr	61.31	108.41	2.46	128.33	24.11	11.02	0.00
N. of dimensions	2				***N. of species***		654
END	3.49						

GSPFF and Lr							
	PC1	*PC2*	*PC3*	*PC4*	*PC5*	*PC6*	*PC7*
Eigenvalues	3.23	1.74	0.66	0.52	0.45	0.22	0.16
Cum. variance	46.27	71.14	80.64	88.04	94.53	97.72	100.00
Loadings	*PC1*	*PC2*	*PC3*	*PC4*	*PC5*	*PC6*	*PC7*
la	0.25	0.75	0.57	0.13	0.06	0.17	0.03
ln	-0.32	0.74	-0.38	-0.32	0.29	0.10	-0.01
ph	0.89	0.23	-0.10	0.17	0.11	-0.12	-0.30
sla	-0.52	0.60	-0.30	0.43	-0.30	-0.04	0.04
ssd	0.84	-0.25	-0.29	0.11	-0.09	0.36	0.03
sm	0.71	0.37	0.01	-0.39	-0.44	-0.08	0.03
Lr	0.89	0.12	-0.11	0.14	0.24	-0.18	0.26
Angles	*la*	*ln*	*ph*	*sla*	*ssd*	*sm*	*Lr*
la	0.00	41.91	56.92	59.70	88.05	44.07	63.59
ln	41.91	0.00	98.83	17.79	129.95	85.98	105.50
ph	56.92	98.83	0.00	116.62	31.12	12.85	6.67
sla	59.70	17.79	116.62	0.00	147.74	103.77	123.29
ssd	88.05	129.95	31.12	147.74	0.00	43.97	24.46
sm	44.07	85.98	12.85	103.77	43.97	0.00	19.52
Lr	63.59	105.50	6.67	123.29	24.46	19.52	0.00
**N. of dimensions**	2				***N. of species***		495
**END**	3.38						

Supplementary Table 9 Principal component analysis (PCA) based on species with complete trait observations, performed on RES traits. ***RES and Dr+Lr*** refers to PCA performed on RES traits along with both Lr and Dr; ***RES***, refers to PCA on RES traits; ***RES and Dr***, refers to PCA on RES traits and maximum rooting depth; ***RES and Lr***, refers to PCA on RES traits and maximum lateral root spread. *Cum. variance,* cumulative variance explained by each axis; *N. of dimensions*, number of relevant dimensions retained; *Angles*, angles across pairs of traits considering all relevant dimensions; *END*, effective number of dimensions; *N. of species*, number of species on which PCA was performed (i.e., species with complete traits observations).

RES and Dr+Lr						
	PC1	*PC2*	*PC3*	*PC4*	*PC5*	*PC6*
Eigenvalues	2.15	1.47	1.09	0.79	0.33	0.12
Cum. variance	36.07	60.68	79.02	92.35	97.96	100.00
Loadings	*PC1*	*PC2*	*PC3*	*PC4*	*PC5*	*PC6*
SRL	0.78	0.54	0.18	0.06	0.07	0.24
D	-0.58	-0.73	0.20	0.22	-0.02	0.22
RTD	-0.48	0.22	-0.68	-0.48	-0.05	0.13
RN	0.32	-0.40	0.47	-0.71	0.01	0.00
Dr	-0.74	0.40	0.31	-0.08	0.42	-0.01
Lr	-0.57	0.54	0.48	-0.04	-0.38	0.01
Angles	***SRL***	***D***	***RTD***	***N***	***Dr***	***Lr***
SRL	0.00	151.36	117.42	79.29	110.71	94.53
D	151.36	0.00	91.22	72.64	76.51	87.60
RTD	117.42	91.22	0.00	159.70	72.81	85.62
RN	79.29	72.64	159.70	0.00	113.69	105.40
Dr	110.71	76.51	72.81	113.69	0.00	17.41
Lr	94.53	87.60	85.62	105.40	17.41	0.00
N. of dimensions	3	***N. of species***	151			
END	4.07					

RES				
	PC1	*PC2*	*PC3*	*PC4*
Eigenvalues	1.94	1.22	0.76	0.07
Cum. variance	48.57	79.10	98.18	100.00
Loadings	*PC1*	*PC2*	*PC3*	*PC4*
SRL	0.82	0.53	0.13	0.17
D	-0.97	-0.12	0.11	0.19
RTD	0.45	-0.71	-0.53	0.09
RN	-0.37	0.64	-0.67	-0.01
Angles	***SRL***	***D***	***RTD***	***N***
SRL	0.00	154.27	90.77	86.57
D	154.27	0.00	114.96	67.70
RTD	90.77	114.96	0.00	177.34
RN	86.57	67.70	177.34	0.00
N. of dimensions	2	***N. of species***	735	
END	2.73			

RES and Dr					
	PC1	*PC2*	*PC3*	*PC4*	*PC5*
Eigenvalues	1.89	1.34	0.84	0.78	0.12
Cum. variance	38.06	65.03	81.94	97.54	100.00
Loadings	*PC1*	*PC2*	*PC3*	*PC4*	*PC5*
SRL	0.93	0.19	0.15	0.15	0.24
D	-0.75	-0.59	0.19	-0.08	0.22
RTD	-0.42	0.65	-0.60	-0.16	0.13
RN	0.23	-0.61	-0.61	0.44	0.00
Dr	-0.50	0.41	0.23	0.73	0.00
Angles	***SRL***	***D***	***RTD***	***N***	***Dr***
SRL	0.00	153.30	111.68	81.00	129.17
D	153.30	0.00	95.02	72.30	77.53
RTD	111.68	95.02	0.00	167.32	17.49
RN	81.00	72.30	167.32	0.00	149.83
Dr	129.17	77.53	17.49	149.83	0.00
N. of dimensions	2	***N. of species***	192		
END	3.69				

RES and Lr					
	PC1	*PC2*	*PC3*	*PC4*	*PC5*
Eigenvalues	1.83	1.31	0.93	0.78	0.12
Cum. variance	36.78	63.11	81.90	97.55	100.00
Loadings	*PC1*	*PC2*	*PC3*	*PC4*	*PC5*
SRL	0.95	0.12	0.11	0.05	0.24
D	-0.79	-0.51	0.15	0.19	0.22
RTD	-0.43	0.62	-0.49	-0.41	0.13
RN	0.21	-0.68	-0.03	-0.69	0.00
Lr	-0.23	0.43	0.81	-0.31	0.00
Angles	***SRL***	***D***	***RTD***	***N***	***Lr***
SRL	0.00	154.58	117.91	79.70	111.30
D	154.58	0.00	87.51	74.88	94.12
RTD	117.91	87.51	0.00	162.39	6.61
RN	79.70	74.88	162.39	0.00	169.00
Lr	111.30	94.12	6.61	169.00	0.00
N. of dimensions	2	***N. of species***	152		
END	3.77				

Supplementary Table 10 Angles correlations across UPFS, GSPFF, and RES trait subsets progressively removing root size traits. *PCA*, correlation coefficients among angles within reduced spaces built considering species with complete traits; *Phyl-PCA*, correlation coefficients among angles within reduced spaces built considering species with complete traits and accounting for phylogenetic relatedness of species. Correlations were computed between UPFS, GSPFF, and RES trait subsets and the ones progressively including root size traits. All p-values are two-sided.

Angles	**PCA** *UPFS*		**Phyl-PCA** *UPFS*	
	r	*p-value*	*r*	*p-value*
UPFS and Dr	0.97	0.000	0.83	0.000
UPFS and Lr	0.95	0.000	0.81	0.000
UPFS and Dr+Lr	0.94	0.000	0.80	0.000
	GSPFF		***GSPFF***	
	r	*p-value*	*r*	*p-value*
GSPFF and Dr	0.99	0.000	0.99	0.000
GSPFF and Lr	0.97	0.000	0.98	0.000
GSPFF and Dr+Lr	0.97	0.000	0.97	0.000
	RES		***RES***	
	r	*p-value*	*r*	*p-value*
RES and Dr	0.95	0.004	0.59	0.219
RES and Lr	0.90	0.015	0.82	0.046
RES and Dr+Lr	0.91	0.012	0.86	0.029

Supplementary Table 11 Angles' correlations among the three different types of reduced spaces across all traits' subsets. *Main – PCA*, correlation coefficients among the main reduced space and PCAs reduced space built considering species with complete set of traits; *Main – PhylPCA*, correlation coefficients among the main reduced space and the phylogenetically informed reduced space built considering species with complete set of traits; *PCA – PhylPCA*, correlation coefficients among PCAs and phylogenetically informed PCAs reduced spaces both built considering species with complete set of traits. All p-values are two-sided.

Angles	**Main - PCA**		**Main - PhylPCA**		**Main - PhylPCA**	
	r	*p-value*	*r*	*p-value*	*r*	*p-value*
**UPFS and Dr+Lr**	0.966	0.000	0.877	0.000	0.911	0.000
**UPFS and Dr**	0.967	0.000	0.868	0.000	0.871	0.000
**UPFS and Lr**	0.963	0.000	0.867	0.000	0.900	0.000
**UPFS**	0.984	0.000	0.949	0.000	0.949	0.000
**GSPFF and Dr+Lr**	0.977	0.000	0.910	0.000	0.947	0.000
**GSPFF and Dr**	0.980	0.000	0.934	0.000	0.932	0.000
**GSPFF and Lr**	0.978	0.000	0.906	0.000	0.944	0.000
**GSPFF**	0.996	0.000	0.940	0.000	0.937	0.000
**RES and Dr+Lr**	0.983	0.000	0.949	0.000	0.955	0.000
**RES and Dr**	0.957	0.000	0.818	0.000	0.868	0.000
**RES and Lr**	0.933	0.000	0.899	0.000	0.954	0.000
**RES**	0.997	0.000	0.994	0.000	0.990	0.000

Supplementary Table 12 Phylogenetically informed principal component analysis (Phyl-PCA) based on species with complete trait observations, performed on UPFS traits. ***UPFS and Dr+Lr*** refers to Phyl-PCA performed on UPFS traits along with both Lr and Dr; ***UPFS***, refers to Phyl-PCA on UPFS traits; ***UPFS and Dr***, refers to Phyl-PCA on UPFS traits and maximum rooting depth; ***UPFS and Lr***, refers to Phyl-PCA on UPFS traits and maximum lateral root spread. *Cum. variance,* cumulative variance explained by each axis; *N. of dimensions*, number of relevant dimensions retained; *Angles*, angles across pairs of traits considering all relevant dimensions; *END*, effective number of dimensions; *N. of species*, number of species on which Phyl-PCA was performed (i.e., species with complete traits observations); *Lambda*, Pagel's lambda.

UPFS and Dr+Lr												
	PC1	*PC2*	*PC3*	*PC4*	*PC5*	*PC6*	*PC7*	*PC8*	*PC9*	*PC10*	*PC11*	*PC12*
Eigenvalues	3.29	2.10	1.82	1.05	0.95	0.67	0.62	0.50	0.31	0.30	0.22	0.17
Cum. variance	27.40	44.91	60.04	68.83	76.75	82.35	87.51	91.65	94.24	96.71	98.56	100.00
Loadings	*PC1*	*PC2*	*PC3*	*PC4*	*PC5*	*PC6*	*PC7*	*PC8*	*PC9*	*PC10*	*PC11*	*PC12*
la	-0.49	0.57	-0.25	-0.27	-0.19	0.13	0.06	0.44	0.03	-0.09	0.19	-0.04
ln	0.10	0.79	-0.09	0.33	-0.15	0.14	0.02	-0.37	0.19	-0.14	0.07	-0.09
ph	-0.78	0.35	0.29	-0.07	-0.01	-0.09	0.10	0.08	0.19	-0.10	-0.31	0.12
sla	0.33	0.68	-0.10	-0.09	-0.17	-0.46	-0.33	-0.02	-0.24	-0.03	-0.05	0.05
ssd	-0.52	-0.41	0.53	0.06	0.13	-0.37	-0.05	-0.03	0.03	-0.29	0.17	-0.07
sm	-0.72	0.20	-0.18	-0.04	0.09	-0.12	0.50	-0.22	-0.28	0.07	0.03	-0.01
SRL	0.45	0.29	0.74	-0.16	0.05	0.14	0.12	-0.05	-0.03	0.00	0.16	0.26
D	-0.35	-0.07	-0.76	0.12	0.42	-0.09	-0.15	-0.05	0.11	-0.03	0.11	0.23
RTD	-0.22	-0.42	-0.13	0.40	-0.76	-0.08	0.07	0.02	0.00	0.01	0.05	0.14
RN	0.25	0.29	0.19	0.78	0.29	-0.10	0.14	0.31	-0.05	0.06	-0.01	-0.01
Dr	-0.71	0.00	0.17	0.24	0.04	0.45	-0.34	-0.04	-0.27	-0.11	-0.04	0.02
Lr	-0.77	0.18	0.35	0.02	-0.02	-0.11	-0.25	-0.07	0.12	0.39	0.11	-0.04
Angles	***la***	***ln***	***ph***	***sla***	***ssd***	***sm***	***SRL***	***D***	***RTD***	***N***	***Dr***	***Lr***
la	0.00	63.05	45.79	65.10	100.29	35.39	105.33	65.49	112.68	107.05	67.62	57.82
ln	63.05	0.00	79.30	34.36	123.73	82.63	79.19	88.38	112.34	50.08	91.05	87.18
ph	45.79	79.30	0.00	93.67	57.89	33.56	91.75	88.55	94.28	96.35	32.54	12.97
sla	65.10	34.36	93.67	0.00	141.70	97.88	66.68	98.26	141.33	73.97	117.86	104.94
ssd	100.29	123.73	57.89	141.70	0.00	72.93	87.81	105.00	63.50	97.58	43.78	45.81
sm	35.39	82.63	33.56	97.88	72.93	0.00	123.74	55.30	80.45	106.00	37.06	38.22
SRL	105.33	79.19	91.75	66.68	87.81	123.74	0.00	164.91	130.32	74.92	109.40	92.62
D	65.49	88.38	88.55	98.26	105.00	55.30	164.91	0.00	62.02	102.37	76.66	90.83
RTD	112.68	112.34	94.28	141.33	63.50	80.45	130.32	62.02	0.00	78.68	61.97	84.30
RN	107.05	50.08	96.35	73.97	97.58	106.00	74.92	102.37	78.68	0.00	86.70	93.88
Dr	67.62	91.05	32.54	117.86	43.78	37.06	109.40	76.66	61.97	86.70	0.00	23.24
Lr	57.82	87.18	12.97	104.94	45.81	38.22	92.62	90.83	84.30	93.88	23.24	0.00
N. of dimensions	4	***Lambda***								***N. of species***		134
END	6.58	0.896										

UPFS										
	PC1	*PC2*	*PC3*	*PC4*	*PC5*	*PC6*	*PC7*	*PC8*	*PC9*	*PC10*
Eigenvalues	2.42	1.94	1.51	1.14	0.89	0.65	0.53	0.50	0.29	0.12
Cum. variance	24.24	43.64	58.71	70.11	79.01	85.52	90.84	95.88	98.78	100.00
Loadings	*PC1*	*PC2*	*PC3*	*PC4*	*PC5*	*PC6*	*PC7*	*PC8*	*PC9*	*PC10*
la	-0.61	0.31	-0.29	-0.12	0.25	0.55	-0.04	0.14	0.21	-0.01
ln	-0.16	0.80	-0.09	-0.09	-0.05	-0.14	0.01	-0.53	0.13	0.02
ph	-0.65	-0.13	-0.62	-0.05	0.04	0.04	0.05	-0.15	-0.38	-0.02
sla	0.02	0.77	-0.10	-0.26	0.04	-0.24	0.38	0.36	-0.06	-0.01
ssd	-0.24	-0.65	-0.48	0.07	-0.17	-0.16	0.39	-0.06	0.26	0.01
sm	-0.72	0.04	-0.19	-0.05	-0.17	-0.41	-0.43	0.21	0.10	0.01
SRL	0.72	0.08	-0.60	0.07	0.15	-0.06	-0.15	0.01	0.06	-0.24
D	-0.69	0.03	0.56	0.38	-0.02	-0.03	0.15	-0.02	0.00	-0.23
RTD	-0.01	-0.21	0.19	-0.85	-0.41	0.11	-0.01	-0.05	0.00	-0.11
RN	0.16	0.35	-0.21	0.41	-0.75	0.24	-0.01	0.09	-0.04	0.01
Angles	***la***	***ln***	***ph***	***sla***	***ssd***	***sm***	***SRL***	***D***	***RTD***	***N***
la	0.00	52.14	37.18	62.12	82.83	24.08	110.23	72.60	90.54	87.25
ln	52.14	0.00	85.22	17.28	129.31	73.72	90.19	86.26	98.55	61.34
ph	37.18	85.22	0.00	92.75	45.98	30.66	97.62	84.54	92.78	94.01
sla	62.12	17.28	92.75	0.00	133.01	84.97	81.24	100.31	87.22	67.44
ssd	82.83	129.31	45.98	133.01	0.00	68.47	84.98	97.02	90.46	105.54
sm	24.08	73.72	30.66	84.97	68.47	0.00	124.89	58.96	89.60	100.03
SRL	110.23	90.19	97.62	81.24	84.98	124.89	0.00	151.68	103.39	58.38
D	72.60	86.26	84.54	100.31	97.02	58.96	151.68	0.00	104.71	95.81
RTD	90.54	98.55	92.78	87.22	90.46	89.60	103.39	104.71	0.00	149.66
RN	87.25	61.34	94.01	67.44	105.54	100.03	58.38	95.81	149.66	0.00
N. of dimensions	4	***Lambda***							***N. of species***	264
END	6.64	0.890								

UPFS and Dr											
	PC1	*PC2*	*PC3*	*PC4*	*PC5*	*PC6*	*PC7*	*PC8*	*PC9*	*PC10*	*PC11*
Eigenvalues	2.78	1.99	1.64	1.05	0.96	0.72	0.57	0.52	0.38	0.24	0.17
Cum. variance	25.26	43.39	58.29	67.81	76.55	83.06	88.22	92.91	96.33	98.47	100.00
Loadings	*PC1*	*PC2*	*PC3*	*PC4*	*PC5*	*PC6*	*PC7*	*PC8*	*PC9*	*PC10*	*PC11*
la	-0.51	-0.60	-0.09	0.14	-0.33	0.06	0.15	0.42	0.02	-0.22	-0.02
ln	0.12	-0.78	-0.15	-0.30	0.07	0.20	-0.17	-0.27	-0.30	-0.11	-0.08
ph	-0.70	-0.21	-0.53	0.06	-0.06	-0.13	0.00	0.09	-0.23	0.32	0.07
sla	0.33	-0.66	0.01	0.07	-0.30	-0.39	0.28	-0.30	0.20	0.06	0.03
ssd	-0.44	0.56	-0.43	0.00	0.04	-0.41	0.17	-0.17	-0.16	-0.22	-0.08
sm	-0.73	-0.22	-0.10	0.02	0.16	-0.20	-0.50	-0.09	0.29	-0.06	0.01
SRL	0.63	0.03	-0.68	0.19	0.00	0.08	-0.11	0.01	0.00	-0.12	0.27
D	-0.49	-0.21	0.65	0.08	0.42	-0.08	0.16	-0.06	-0.14	-0.07	0.23
RTD	-0.26	0.30	0.22	-0.66	-0.57	-0.01	-0.08	-0.04	-0.02	-0.02	0.15
RN	0.32	-0.19	-0.26	-0.65	0.46	-0.22	0.13	0.27	0.10	0.02	0.00
Dr	-0.62	0.06	-0.37	-0.15	0.13	0.48	0.32	-0.23	0.23	0.00	0.02
Angles	***la***	***ln***	***ph***	***sla***	***ssd***	***sm***	***SRL***	***D***	***RTD***	***N***	***Dr***
la	0.00	56.59	42.29	66.19	96.72	33.71	109.16	60.59	103.86	100.13	60.33
ln	56.59	0.00	79.20	32.69	126.78	81.99	82.52	91.33	98.56	51.60	91.79
ph	42.29	79.20	0.00	97.32	56.70	28.88	94.79	86.18	92.66	96.63	24.40
sla	66.19	32.69	97.32	0.00	146.88	99.35	73.61	90.97	123.53	71.64	117.39
ssd	96.72	126.78	56.70	146.88	0.00	68.32	87.44	105.20	73.78	102.19	41.45
sm	33.71	81.99	28.88	99.35	68.32	0.00	122.94	57.85	81.12	106.75	33.48
SRL	109.16	82.52	94.79	73.61	87.44	122.94	0.00	157.93	124.62	70.87	103.57
D	60.59	91.33	86.18	90.97	105.20	57.85	157.93	0.00	76.88	119.83	86.08
RTD	103.86	98.56	92.66	123.53	73.78	81.12	124.62	76.88	0.00	68.41	70.61
RN	100.13	51.60	96.63	71.64	102.19	106.75	70.87	119.83	68.41	0.00	91.77
Dr	60.33	91.79	24.40	117.39	41.45	33.48	103.57	86.08	70.61	91.77	0.00
N. of dimensions	4	***Lambda***								**N. of species**	158
END	6.82	0.893									

UPFS and Lr											
	PC1	*PC2*	*PC3*	*PC4*	*PC5*	*PC6*	*PC7*	*PC8*	*PC9*	*PC10*	*PC11*
Eigenvalues	2.89	2.09	1.79	1.02	0.95	0.64	0.50	0.42	0.29	0.23	0.17
Cum. variance	26.27	45.30	61.59	70.90	79.50	85.34	89.87	93.68	96.36	98.41	100.00
Loadings	*PC1*	*PC2*	*PC3*	*PC4*	*PC5*	*PC6*	*PC7*	*PC8*	*PC9*	*PC10*	*PC11*
la	0.52	-0.57	0.20	0.23	-0.22	0.14	0.44	-0.02	0.08	-0.20	-0.04
ln	-0.14	-0.78	0.13	-0.32	-0.12	0.12	-0.35	0.22	0.19	-0.07	-0.09
ph	0.77	-0.35	-0.35	-0.02	0.00	0.04	0.07	0.04	0.17	0.32	0.13
sla	-0.29	-0.68	0.11	0.06	-0.17	-0.58	-0.03	-0.26	-0.04	0.03	0.04
ssd	0.50	0.41	-0.57	-0.13	0.16	-0.28	-0.05	-0.12	0.28	-0.18	-0.08
sm	0.77	-0.20	0.11	-0.03	0.11	0.28	-0.28	-0.40	-0.15	-0.04	-0.01
SRL	-0.50	-0.29	-0.72	0.17	0.02	0.17	-0.06	-0.02	-0.02	-0.16	0.26
D	0.39	0.07	0.74	-0.05	0.42	-0.16	-0.04	0.13	0.07	-0.10	0.23
RTD	0.21	0.42	0.13	-0.51	-0.69	0.00	0.01	-0.04	-0.01	-0.04	0.14
RN	-0.30	-0.28	-0.15	-0.74	0.40	0.06	0.29	-0.10	-0.07	0.01	-0.01
Lr	0.73	-0.17	-0.40	-0.07	-0.01	-0.23	-0.03	0.31	-0.33	-0.07	-0.03
Angles	***la***	***ln***	***ph***	***sla***	***ssd***	***sm***	***SRL***	***D***	***RTD***	***N***	***Lr***
la	0.00	62.96	46.09	64.52	98.97	37.31	105.36	64.12	111.98	105.67	57.51
ln	62.96	0.00	80.56	28.92	123.79	83.82	79.38	89.52	106.70	49.86	89.32
ph	46.09	80.56	0.00	92.17	56.02	31.46	92.54	88.60	91.89	94.87	12.25
sla	64.52	28.92	92.17	0.00	138.89	97.61	67.01	97.38	132.81	69.86	102.95
ssd	98.97	123.79	56.02	138.89	0.00	69.07	88.72	105.34	64.25	96.76	44.02
sm	37.31	83.82	31.46	97.61	69.07	0.00	122.96	57.22	79.13	103.69	35.78
SRL	105.36	79.38	92.54	67.01	88.72	122.96	0.00	163.19	127.52	74.96	92.89
D	64.12	89.52	88.60	97.38	105.34	57.22	163.19	0.00	67.29	106.27	91.75
RTD	111.98	106.70	91.89	132.81	64.25	79.13	127.52	67.29	0.00	72.44	83.55
RN	105.67	49.86	94.87	69.86	96.76	103.69	74.96	106.27	72.44	0.00	94.16
Lr	57.51	89.32	12.25	102.95	44.02	35.78	92.89	91.75	83.55	94.16	0.00
N. of dimensions	4	***Lambda***								***N. of species***	135
END	6.40	0.900									

Supplementary Table 13 Phylogenetically informed principal component analysis (Phyl-PCA) based on species with complete trait observations, performed on GSPFF traits. ***GSPFF and Dr+Lr*** refers to Phyl-PCA performed on GSPFF traits along with both Lr and Dr; ***GSPFF***, refers to Phyl-PCA on GSPFF traits; ***GSPFF and Dr***, refers to Phyl-PCA on GSPFF traits and maximum rooting depth; ***GSPFF and Lr***, refers to Phyl-PCA on GSPFF traits and maximum lateral root spread. *Cum. variance,* cumulative variance explained by each axis; *N. of dimensions*, number of relevant dimensions retained; *Angles*, angles across pairs of traits considering all relevant dimensions; *END*, effective number of dimensions; *N. of species*, number of species on which Phyl-PCA was performed (i.e., species with complete traits observations); *Lambda*, Pagel's lambda.

GSPFF and Dr+Lr								
	PC1	*PC2*	*PC3*	*PC4*	*PC5*	*PC6*	*PC7*	*PC8*
Eigenvalues	2.70	1.91	0.83	0.75	0.65	0.45	0.43	0.29
Cum. variance	33.70	57.56	67.96	77.35	85.44	91.04	96.36	100.00
Loadings	*PC1*	*PC2*	*PC3*	*PC4*	*PC5*	*PC6*	*PC7*	*PC8*
la	0.47	0.62	0.44	-0.12	0.26	0.12	0.05	0.30
ln	-0.04	0.74	-0.39	-0.05	-0.46	0.29	0.02	0.07
ph	0.80	0.25	-0.11	-0.03	0.29	0.23	-0.25	-0.31
sla	-0.31	0.68	-0.41	0.13	0.31	-0.37	-0.11	0.06
ssd	0.47	-0.62	-0.44	0.24	0.14	0.16	-0.09	0.29
sm	0.58	0.21	0.22	0.70	-0.24	-0.13	0.01	-0.04
Dr	0.75	-0.10	0.01	-0.37	-0.32	-0.31	-0.29	0.08
Lr	0.80	0.00	-0.25	-0.16	0.05	-0.14	0.50	-0.07
Angles	***la***	***ln***	***ph***	***sla***	***ssd***	***sm***	***Dr***	***Lr***
la	0.00	40.39	35.83	61.24	106.15	32.94	60.38	53.29
ln	40.39	0.00	76.22	20.85	146.55	73.33	100.77	93.68
ph	35.83	76.22	0.00	97.07	70.32	2.89	24.55	17.46
sla	61.24	20.85	97.07	0.00	167.40	94.18	121.62	114.53
ssd	106.15	146.55	70.32	167.40	0.00	73.21	45.78	52.86
sm	32.94	73.33	2.89	94.18	73.21	0.00	27.44	20.35
Dr	60.38	100.77	24.55	121.62	45.78	27.44	0.00	7.09
Lr	53.29	93.68	17.46	114.53	52.86	20.35	7.09	0.00
N. of dimensions	2	***Lambda***		***N. of species***	494			
END	4.90	0.902						

GSPFF						
	PC1	*PC2*	*PC3*	*PC4*	*PC5*	*PC6*
Eigenvalues	1.79	1.75	0.76	0.74	0.56	0.40
Cum. variance	29.91	58.99	71.61	84.01	93.36	100.00
Loadings	*PC1*	*PC2*	*PC3*	*PC4*	*PC5*	*PC6*
la	-0.80	0.03	-0.14	-0.41	0.23	0.34
ln	-0.41	0.58	-0.01	0.62	0.33	0.01
ph	-0.74	-0.39	-0.33	0.00	-0.05	-0.42
sla	-0.38	0.71	-0.06	0.02	-0.59	0.07
ssd	-0.05	-0.78	-0.26	0.43	-0.21	0.31
sm	-0.53	-0.38	0.75	0.08	-0.09	-0.01
Angles	***la***	***ln***	***ph***	***sla***	***ssd***	***sm***
la	0.00	52.48	29.76	59.58	88.49	37.90
ln	52.48	0.00	82.24	7.11	140.97	90.38
ph	29.76	82.24	0.00	89.35	58.73	8.14
sla	59.58	7.11	89.35	0.00	148.08	97.49
ssd	88.49	140.97	58.73	148.08	0.00	50.59
sm	37.90	90.38	8.14	97.49	50.59	0.00
N. of dimensions	2	***Lambda***	***N. of species***	1733		
END	4.58	0.932				

GSPFF and Dr							
	PC1	*PC2*	*PC3*	*PC4*	*PC5*	*PC6*	*PC7*
Eigenvalues	2.11	1.80	0.81	0.73	0.71	0.52	0.32
Cum. variance	30.11	55.87	67.44	77.91	87.99	95.40	100.00
Loadings	*PC1*	*PC2*	*PC3*	*PC4*	*PC5*	*PC6*	*PC7*
la	0.32	-0.71	-0.40	0.22	-0.29	-0.10	0.29
ln	-0.27	-0.68	0.51	-0.11	0.17	-0.42	0.04
ph	0.72	-0.44	-0.04	-0.27	-0.29	-0.03	-0.37
sla	-0.49	-0.60	0.05	-0.40	0.00	0.48	0.08
ssd	0.63	0.43	0.07	-0.57	0.00	-0.10	0.28
sm	0.59	-0.31	-0.20	0.06	0.71	0.09	-0.02
Dr	0.65	-0.03	0.59	0.34	-0.12	0.29	0.09
Angles	***la***	***ln***	***ph***	***sla***	***ssd***	***sm***	***Dr***
la	0.00	45.55	34.46	63.42	100.22	38.33	62.95
ln	45.55	0.00	80.01	17.87	145.77	83.88	108.50
ph	34.46	80.01	0.00	97.88	65.75	3.86	28.49
sla	63.42	17.87	97.88	0.00	163.64	101.75	126.37
ssd	100.22	145.77	65.75	163.64	0.00	61.89	37.26
sm	38.33	83.88	3.86	101.75	61.89	0.00	24.63
Dr	62.95	108.50	28.49	126.37	37.26	24.63	0.00
N. of dimensions	2	***Lambda***		N. of species	654		
END	5.02	0.915					

GSPFF and Lr							
	PC1	*PC2*	*PC3*	*PC4*	*PC5*	*PC6*	*PC7*
Eigenvalues	2.26	1.89	0.83	0.71	0.57	0.44	0.30
Cum. variance	32.26	59.30	71.20	81.33	89.50	95.75	100.00
Loadings	*PC1*	*PC2*	*PC3*	*PC4*	*PC5*	*PC6*	*PC7*
la	0.57	-0.54	0.44	0.27	0.07	-0.05	0.32
ln	0.04	-0.74	-0.39	-0.18	-0.48	-0.16	0.08
ph	0.84	-0.12	-0.11	0.21	0.12	-0.34	-0.30
sla	-0.18	-0.72	-0.41	-0.02	0.51	0.15	0.02
ssd	0.40	0.70	-0.43	-0.10	0.14	-0.19	0.31
sm	0.64	-0.10	0.23	-0.71	0.09	0.09	-0.04
Lr	0.78	0.12	-0.25	0.22	-0.19	0.48	-0.03
Angles	***la***	***ln***	***ph***	***sla***	***ssd***	***sm***	***Lr***
la	0.00	43.21	35.45	60.54	103.70	34.40	52.45
ln	43.21	0.00	78.66	17.33	146.91	77.61	95.66
ph	35.45	78.66	0.00	95.99	68.25	1.05	16.99
sla	60.54	17.33	95.99	0.00	164.24	94.94	112.99
ssd	103.70	146.91	68.25	164.24	0.00	69.30	51.26
sm	34.40	77.61	1.05	94.94	69.30	0.00	18.05
Lr	52.45	95.66	16.99	112.99	51.26	18.05	0.00
N. of dimensions	2	***Lambda***	***N. of species***	495			
END	4.67	0.908					

Supplementary Table 14 Phylogenetically informed principal component analysis (Phyl-PCA) based on species with complete trait observations, performed on RES traits. ***RES and Dr+Lr*** refers to Phyl-PCA performed on RES traits along with both Lr and Dr; ***RES***, refers to Phyl-PCA on RES traits; ***RES and Dr***, refers to Phyl-PCA on RES traits and maximum rooting depth; ***RES and Lr***, refers to Phyl-PCA on RES traits and maximum lateral root spread. *Cum. variance,* cumulative variance explained by each axis; *N. of dimensions*, number of relevant dimensions retained; *Angles*, angles across pairs of traits considering all relevant dimensions; *END*, effective number of dimensions; *N. of species*, number of species on which Phyl-PCA was performed (i.e., species with complete trait observations); *Lambda*, Pagel's lambda.

**RES and Dr+Lr**						
	PC1	PC2	PC3	PC4	PC5	PC6
Eigenvalues	2.17	1.31	1.03	0.90	0.43	0.16
Cum. variance	36.10	57.95	75.06	90.12	97.31	100.00
Loadings	PC1	PC2	PC3	PC4	PC5	PC6
SRL	0.85	0.41	0.02	0.17	-0.04	-0.29
D	-0.71	-0.42	-0.50	-0.05	0.05	-0.25
RTD	-0.43	-0.04	0.80	-0.39	0.04	-0.14
RN	0.34	0.24	-0.35	-0.83	0.05	0.02
Dr	-0.62	0.62	-0.09	-0.02	-0.48	-0.01
Lr	-0.50	0.73	-0.07	0.15	0.44	0.01
Angles	SRL	D	RTD	N	Dr	Lr
SRL	0.00	149.94	114.85	42.20	109.73	99.14
D	149.94	0.00	95.20	108.97	74.60	84.29
RTD	114.85	95.20	0.00	151.31	77.53	80.65
RN	42.20	108.97	151.31	0.00	93.84	86.48
Dr	109.73	74.60	77.53	93.84	0.00	10.65
Lr	99.14	84.29	80.65	86.48	10.65	0.00
N. of dimensions	3					
END	4.24		N. of species			151
Lambda	0.834					

**RES**				
	PC1	PC2	PC3	PC4
Eigenvalues	1.78	1.31	0.80	0.10
Cum. variance	44.53	77.37	97.43	100.00
Loadings	PC1	PC2	PC3	PC4
SRL	0.92	0.28	-0.17	-0.21
D	-0.96	0.18	-0.06	-0.22
RTD	0.13	-0.83	0.53	-0.11
RN	0.03	0.71	0.70	0.01
Angles	SRL	D	RTD	N
SRL	0.00	152.06	98.16	70.25
D	152.06	0.00	109.77	81.81
RTD	98.16	109.77	0.00	168.41
RN	70.25	81.81	168.41	0.00
N. of dimensions	2			
END	2.88	N. of species		735
Lambda	0.715			

**RES and Dr**					
	PC1	PC2	PC3	PC4	PC5
Eigenvalues	1.99	1.06	0.92	0.87	0.16
Cum. variance	39.83	60.94	79.38	96.77	100.00
Loadings	PC1	PC2	PC3	PC4	PC5
SRL	0.92	0.07	-0.05	0.25	-0.28
D	-0.78	-0.55	0.12	-0.10	-0.25
RTD	-0.43	0.82	-0.03	-0.36	-0.14
RN	0.41	-0.05	0.82	-0.41	0.01
Dr	-0.43	0.29	0.49	0.71	0.00
Angles	SRL	D	RTD	N	Dr
SRL	0.00	149.09	113.45	11.34	142.16
D	149.09	0.00	97.46	137.75	68.76
RTD	113.45	97.46	0.00	124.79	28.70
RN	11.34	137.75	124.79	0.00	153.50
Dr	142.16	68.76	28.70	153.50	0.00
N. of dimensions	2				
END	3.72		N. of species		192
Lambda	0.744				

**RES and Lr**					
	PC1	PC2	PC3	PC4	PC5
Eigenvalues	1.94	1.04	0.95	0.91	0.16
Cum. variance	38.76	59.65	78.60	96.72	100.00
Loadings	PC1	PC2	PC3	PC4	PC5
SRL	0.93	0.10	0.11	0.15	-0.29
D	-0.80	-0.54	0.09	-0.07	-0.25
RTD	-0.43	0.74	-0.37	-0.33	-0.14
RN	0.41	-0.24	0.11	-0.87	0.02
Lr	-0.28	0.38	0.88	-0.04	0.00
Angles	SRL	D	RTD	N	Lr
SRL	0.00	152.00	114.42	36.15	120.11
D	152.00	0.00	93.58	115.84	87.90
RTD	114.42	93.58	0.00	150.58	5.68
RN	36.15	115.84	150.58	0.00	156.26
Lr	120.11	87.90	5.68	156.26	0.00
N. of dimensions	2				
END	3.79	N. of species			152
Lambda	0.856				
