# Supplementary material for: Aboveground and belowground sizes are aligned in the unified spectrum of plant form and function
Source: Nat Commun. 2024 Oct 24;15:9199. doi: 10.1038/s41467-024-53180-x (PMC11502772; doi:10.1038/s41467-024-53180-x)
Supplement: Supplementary file 2 — Reporting Summary [file 41467_2024_53180_MOESM2_ESM.pdf]

nature portfolio

Corresponding author(s): Eleonora Beccari

Last updated by author(s): Sep 24, 2024

Reporting Summary

Nature Portfolio wishes to improve the reproducibility of the work that we publish. This form provides structure for consistency and transparency in reporting. For further information on Nature Portfolio policies, see our Editorial Policies and the Editorial Policy Checklist.

Statistics

For all statistical analyses, confirm that the following items are present in the figure legend, table legend, main text, or Methods section.

n/a	Confirmed	
☐	☒	The exact sample size (*n*) for each experimental group/condition, given as a discrete number and unit of measurement
☐	☒	A statement on whether measurements were taken from distinct samples or whether the same sample was measured repeatedly
☐	☒	The statistical test(s) used AND whether they are one- or two-sided<br>*Only common tests should be described solely by name; describe more complex techniques in the Methods section.*
☐	☒	A description of all covariates tested
☐	☒	A description of any assumptions or corrections, such as tests of normality and adjustment for multiple comparisons
☐	☒	A full description of the statistical parameters including central tendency (e.g. means) or other basic estimates (e.g. regression coefficient) AND variation (e.g. standard deviation) or associated estimates of uncertainty (e.g. confidence intervals)
☐	☒	For null hypothesis testing, the test statistic (e.g. *F*, *t*, *r*) with confidence intervals, effect sizes, degrees of freedom and *P* value noted<br>*Give P values as exact values whenever suitable.*
☒	☐	For Bayesian analysis, information on the choice of priors and Markov chain Monte Carlo settings
☒	☐	For hierarchical and complex designs, identification of the appropriate level for tests and full reporting of outcomes
☐	☒	Estimates of effect sizes (e.g. Cohen's *d*, Pearson's *r*), indicating how they were calculated

Our web collection on statistics for biologists contains articles on many of the points above.

Software and code

Policy information about availability of computer code

Data collection	Phylogenetic information for all considered species was downloaded from the tree available in the R package ‘V.PhyloMaker2’ version 0.1.0 (Jin, 2023).
Data analysis	All analyses were performed using R version 4.0.3. (R Core Team 2020). Nomenclatures of species was homogenized using WorldFlora’ version 2023.01 (Kindt, 2020) and ‘Taxonstand’ version 2.4 (Cayuela et al., 2021) packages. Effective number of dimensions was extracted using ‘vegan’ package version 2.5-7 (Oksanen et al., 2020). The phylogenetically-informed PCAs were performed using ‘phytools’ R package version 0.7-47 (Revell, 2012). Data and codes required to replicate the analysis can be found in Figshare (https://figshare.com/s/126110f6a3aa435a1871).

For manuscripts utilizing custom algorithms or software that are central to the research but not yet described in published literature, software must be made available to editors and reviewers. We strongly encourage code deposition in a community repository (e.g. GitHub). See the Nature Portfolio guidelines for submitting code & software for further information.

Data

Policy information about availability of data

All manuscripts must include a data availability statement. This statement should provide the following information, where applicable:

- Accession codes, unique identifiers, or web links for publicly available datasets
- A description of any restrictions on data availability
- For clinical datasets or third party data, please ensure that the statement adheres to our policy

The data used in the analysis can be found on Figshare at https://figshare.com/s/126110f6a3aa435a1871. TRY data can be accessed at https://www.try-db.org/TryWeb/Home.php. Original GRooT data are available at https://doi.org/10.1111/geb.13179. Original root size data can be found at https://doi.org/10.1111/nph.18031. Data on species nomenclature are available at https://www.worldfloraonline.org. Plant phylogenies can be accessed at https://doi.org/10.1016/j.pld.2022.05.005

Research involving human participants, their data, or biological material

Policy information about studies with human participants or human data. See also policy information about sex, gender (identity/presentation), and sexual orientation and race, ethnicity and racism.

Reporting on sex and gender	Not applicable
Reporting on race, ethnicity, or other socially relevant groupings	Not applicable
Population characteristics	Not applicable
Recruitment	Not applicable
Ethics oversight	Not applicable

Note that full information on the approval of the study protocol must also be provided in the manuscript.

Field-specific reporting

Please select the one below that is the best fit for your research. If you are not sure, read the appropriate sections before making your selection.

☐ Life sciences ☐ Behavioural & social sciences ☒ Ecological, evolutionary & environmental sciences

For a reference copy of the document with all sections, see nature.com/documents/nr-reporting-summary-flat.pdf

Ecological, evolutionary & environmental sciences study design

All studies must disclose on these points even when the disclosure is negative.

Study description

We collected aboveground, fine roots, and belowground size plant traits information from public databases (total of twelve traits). We calculated Pearson correlation coefficients across the full set of twelve traits. Then we tested whether the inclusion of root size traits affect dimensionality and the correlations structures defining the main dimensions of trait variation observed in the original UPFS (Unified Plant Functional Spectrum as described in Carmona et al. 2021) For that, we performed a series of eigendecompositions using traits correlation matrix and progressively including root size traits to the set of aboveground and fine root traits defining the UPFS. To extract the relevant dimension of traits variation, for each eigendecomposition, we estimated traits loadings of the resulting space. To understand the relationships among traits in each space, we estimated the correlations between trait loadings considering all relevant dimensions in the space. To test whether the inclusion of root size traits influences traits correlations observed in the UPFS space of Carmona et al. 2021, we compared the traits angles across the spaces built by performing an eigendecomposition progressively including root size traits to UPFS. Additionally, for each eigendecomposition, we calculated the effective number of dimensions which we compared to the effective number of dimensions expected if an uncorrelated trait is added to the set of ten traits defining UPFS. We performed the same set of analyses by progressively including root size traits also to the subset of aboveground traits and belowground fine root traits separately. Additionally, we tested whether the abovementioned spaces remained consistent when considering only woody and herbaceous species subsets, when considering only complete traits information, and when considering for species relatedness. We collected above- and belowground plant traits information from public databases. We performed a series of eigendecompositions progressively including root size traits to the set of aboveground and fine root traits defining the Unified Plant Functional Spectrum (UPFS as described in Carmona et al. 2021). To extract the relevant dimension of traits variation, for each eigendecomposition, we estimated traits loadings of the resulting space. To understand the relationships among traits in each space, we estimated the correlations between trait loadings considering all relevant dimensions in the space. To test whether the inclusion of root size traits influences traits correlations observed in the UPFS space of Carmona et al. 2021, we compared the traits angles across the spaces built by performing an eigendecomposition progressively including root size traits to UPFS. Additionally, for each eigendecomposition, we calculated the effective number of dimensions which we compared to the effective number of dimensions expected if an uncorrelated trait is added to the set of ten traits defining UPFS. We performed

	the same set of analyses by progressively including root size traits also to the subset of aboveground traits and belowground fine root traits separately. Additionally, we tested whether the abovementioned spaces remained consistent when considering only woody and herbaceous species subsets, when considering only complete traits information, and when considering for species relatedness.
Research sample	We extracted aboveground (six traits, Kattge et al., 2020) , fine roots (four traits, Guerrero-Ramírez et al., 2021), and root size traits (two traits, Tumber-Dávila et al. 2022) information for 39,334 species. The dataset contained 49.47% of woody species and 32.04% herbaceous species (with 18.29% of species missing woodiness information). The dataset showed different level of traits completeness (i.e. NAs were present) depending on the subset of trait considered. Specifically, pairs of traits with common observations ranged from a minimum of 257 common species (to a maximum of 7,217. The trait with the highest completeness had observations for the 58.48% of total species, whereas the least complete trait had observations for 3.13% of total species.
Sampling strategy	We used all information available (see above).
Data collection	Data was primarily collected from the aforementioned databases by Eleonora Beccari with assistance from Carlos P. Carmona
Timing and spatial scale	Trait information and phylogeny were collected between June 2022 and February 2023.
Data exclusions	For each trait, outlier values were checked on literature and set as NA if considered biased
Reproducibility	All our data is based on computer analyses. The code to reproduce the results will be made available in case of paper acceptance. Since null models are involved, exact results might not be reproduced, but conclusions should remain very stable.
Randomization	This is not relevant for our study since we do not perform any experiment
Blinding	Blinding is not relevant for our study since we collected information from databases and published papers

Did the study involve field work? ☐ Yes ☒ No

Reporting for specific materials, systems and methods

We require information from authors about some types of materials, experimental systems and methods used in many studies. Here, indicate whether each material, system or method listed is relevant to your study. If you are not sure if a list item applies to your research, read the appropriate section before selecting a response.

Materials & experimental systems

n/a	Involved in the study
☒	☐ Antibodies
☒	☐ Eukaryotic cell lines
☒	☐ Palaeontology and archaeology
☒	☐ Animals and other organisms
☒	☐ Clinical data
☒	☐ Dual use research of concern
☒	☐ Plants

Methods

n/a	Involved in the study
☒	☐ ChIP-seq
☒	☐ Flow cytometry
☒	☐ MRI-based neuroimaging

Plants

Seed stocks	Not applicable
Novel plant genotypes	Not applicable
Authentication	Not applicable
